# A Blockwise Bootstrap-Based Two-Sample Test for High-Dimensional Time Series

**DOI:** 10.3390/e26030226

**Published:** 2024-03-01

**Authors:** Lin Yang

**Affiliations:** Joint Laboratory of Data Science and Business Intelligence, Southwestern University of Finance and Economics, Chengdu 611130, China; yanglin@smail.swufe.edu.cn

**Keywords:** two-sample testing, high-dimensional time series, α-mixing, Gaussian approximation, blockwise bootstrap

## Abstract

We propose a two-sample testing procedure for high-dimensional time series. To obtain the asymptotic distribution of our $\ell_{\infty }$-type test statistic under the null hypothesis, we establish high-dimensional central limit theorems (HCLTs) for an α-mixing sequence. Specifically, we derive two HCLTs for the maximum of a sum of high-dimensional α-mixing random vectors under the assumptions of bounded finite moments and exponential tails, respectively. The proposed HCLT for α-mixing sequence under bounded finite moments assumption is novel, and in comparison with existing results, we improve the convergence rate of the HCLT under the exponential tails assumption. To compute the critical value, we employ the blockwise bootstrap method. Importantly, our approach does not require the independence of the two samples, making it applicable for detecting change points in high-dimensional time series. Numerical results emphasize the effectiveness and advantages of our method.

## 1. Introduction

A fundamental testing problem in multivariate analysis involves assessing the equality of two mean vectors, denoted as $\mu_{X}$ and $\mu_{Y}$. Since its inception by [1], the Hotelling $T^{2}$ test has proven to be a valuable tool in multivariate analyses. Subsequently, numerous studies have addressed the testing of $\mu_{X}=\mu_{Y}$, within various contexts and under distinct assumptions. See refs. [2,3], along with their respective references.

Consider two sets of observations, ${\left\{X_{t}\right\}}_{t=1}^{n_{1}}$ and ${\left\{Y_{t}\right\}}_{t=1}^{n_{2}}$, where $X_{t}={\left(X_{t,1},\ldots ,X_{t,p}\right)}^{T}$ and $Y_{t}={\left(Y_{t,1},\ldots ,Y_{t,p}\right)}^{T}$. These observations are drawn from two populations with means $\mu_{X}$ and $\mu_{Y}$, respectively. The classical test aims to test the hypotheses:

(1) $$\begin{array}{c} H_{0}:\mu_{X}=\mu_{Y}\;\;\;\;\mathrm{versus}\;\;\;\;H_{1}:\mu_{X}\neq \mu_{Y}. \end{array}$$

When ${\left\{X_{t}\right\}}_{t=1}^{n_{1}}$ and ${\left\{Y_{t}\right\}}_{t=1}^{n_{2}}$ are two independent sequences and independent with each other, a considerable body of literature focuses on testing Hypothesis (1). The $\ell_{2}$-type test statistic corresponding to (1) is of the form ${\left(\bar{X}-\bar{Y}\right)}^{T}S^{-1}\left(\bar{X}-\bar{Y}\right)$, where $\bar{X}=n_{1}^{-1}\sum_{t=1}^{n_{1}}X_{t}$, $\bar{Y}=n_{2}^{-1}\sum_{t=1}^{n_{2}}Y_{t}$ and $S^{-1}$ is the weight matrix. A straightforward choice for $S^{-1}$ is the identity matrix $I_{p}$ [4,5], implying equal weighting for each dimension. Several classical asymptotic theories have been developed based on this selection of $S^{-1}$. However, this choice disregards the variability in each dimension and the correlations between them, resulting in suboptimal performance, particularly in the presence of heterogeneity or the existence of correlations between dimensions. In recent decades, numerous researchers have investigated various choices for $S^{-1}$ along with the corresponding asymptotic theories. See refs. [6,7]. In addition, some researchers have developed a framework centered on $\ell_{\infty }$-type test statistics, represented as $\max_{j\in [p]}\left|{\left(S^{-1/2}\left(\bar{X}-\bar{Y}\right)\right)}_{j}\right|$ [8,9,10]. Extreme value theory plays a pivotal role in deriving the asymptotic behaviors of these test statistics.

However, when ${\left\{X_{t}\right\}}_{t=1}^{n_{1}}$ and ${\left\{Y_{t}\right\}}_{t=1}^{n_{2}}$ are two weakly dependent sequences and are not independent of each other, the above methods may not work well. In this paper, we introduce an $\ell_{\infty }$-type test statistic $T_{n}:={\left(n_{1}n_{2}\right)}^{1/2}{\left(n_{1}+n_{2}\right)}^{-1/2}{\left|\bar{X}-\bar{Y}\right|}_{\infty }$ for testing $H_{0}$ under two dependent sequences. Based on Σ, which represents the variance of ${\left(n_{1}n_{2}\right)}^{1/2}{\left(n_{1}+n_{2}\right)}^{-1/2}\left(\bar{X}-\bar{Y}\right)$, we construct a Gaussian maxima, denoted as $T_{n}^{G}$, to approximate $T_{n}$ under the null hypothesis. When $n_{1}=n_{2}=n$, $T_{n}$ can be written as $|S_{n}{|}_{\infty }$, the maximum of a sum of high-dimensional weakly dependent random vectors, where $S_{n}=n^{-1/2}\sum_{t=1}^{n}\left(X_{t}-Y_{t}\right)$. Let $T_{n}^{G}={\left|G\right|}_{\infty }$ with $G={\left(G_{1},\ldots ,G_{p}\right)}^{T}$∼$N\{0,\mathrm{var}\left(S_{n}\right)\}$ and A be a class of Borel subsets in $R^{p}$. Define

$$\rho_{n}\left(A\right)=\underset{A\in A}{\sup}\left|P\left(S_{n}\in A\right)-P\left(G\in A\right)\right|.$$

Paticularly, let $A^{\max}$ consists of all sets $A^{\max}$ of the form $A^{\max}=\{{\left(a_{1},\ldots ,a_{p}\right)}^{T}\in R^{p}$: $\max_{j\in [p]}|a_{j}\left|\leq x\right\}$ with some x∈R. Then we have

$$\begin{array}{c} \rho_{n}\left(A^{\max}\right)=\underset{x\in R}{\sup}\left|P\left(T_{n}\leq x\right)-P\left(T_{n}^{G}\leq x\right)\right|. \end{array}$$

Note that $\rho_{n}\left(A^{\max}\right)$ is the Kolmogorov distance between $T_{n}$ and $T_{n}^{G}$.

When dimension *p* diverges exponentially with respect to the sample size *n*, several studies have focused on deriving $\rho_{n}\left(A^{\max}\right)=o\left(1\right)$ under a weakly dependent assumption. Based on the coupling method for β-mixing sequence, ref. [11] obtained $\rho_{n}\left(A^{\max}\right)=o\left(1\right)$ under the β-mixing condition, contributing to the understanding of such phenomena. Ref. [12] extended the scope of the investigation to the physical dependence framework introduced by [13]. Considering three distinct types of dependence—namely α-mixing, *m*-dependence, and physical dependence measures—ref. [14] made significant strides. They established nonasymptotic error bounds for Gaussian approximations of sums involving high-dimensional dependent random vectors. Their analysis encompassed various scenarios of A, including hyper-rectangles, simple convex sets, and sparsely convex sets. Let $A^{\mathrm{re}}$ be the class of all hyper-rectangles in $R^{p}$. Under the α-mixing scenario and some mild regularity conditions, [14] showed

$$\rho_{n}\left(A^{\mathrm{re}}\right)≲\frac{{\left\{\log\left(pn\right)\right\}}^{7/6}}{n^{1/9}},$$

hence the Gaussian approximation holds if $\log\left(pn\right)=o\left(n^{2/21}\right)$. In this paper, under some conditions similar to or even weaker than [14], we obtain

$$\rho_{n}\left(A^{\max}\right)≲\frac{{\left\{\log\left(pn\right)\right\}}^{3/2}}{n^{1/6}},$$

which implies the Gaussian approximation holds if $\log\left(pn\right)=o\left(n^{1/9}\right)$. Refer to Remark 1 for more details on the comparison of the convergence rates. By using the Gaussian-to-Gaussian comparison and Nazarov’s inequality for *p*-dimensional random vectors, we can easily extend our result to $\rho_{n}\left(A^{\mathrm{re}}\right)≲{\left\{\log\left(pn\right)\right\}}^{3/2}n^{-1/6}$. Given that our framework and numerous testing procedures rely on $\ell_{\infty }$-type test statistics, we thus propose our results under $A^{\max}$. When *p* diverges polynomially with respect to *n*, to the best of our knowledge, there is no existing literature providing the convergence rate of $\rho_{n}\left(A^{\max}\right)$ for α-mixing sequences under bounded finite moments.

Based on the Gaussian approximation for high-dimensional independent random vectors [15,16], we employ the coupling method for α-mixing sequence [17] and “big-and-small” block technique to specify the convergence rate of $\rho_{n}\left(A^{\max}\right)$ under various divergence rates of *p*. For more details, refer to Theorem 1 in Section 3.1 and its corresponding proof in Appendix A. Given that Σ is typically unknown in practice, we develop a data-driven procedure based on blockwise wild bootstrap [18] to determine the critical value for a given significance level α. The blockwise wild bootstrap method is widely used in the time series analysis. See [19,20] and references within.

The independence between ${\left\{X_{t}\right\}}_{t=1}^{n_{1}}$ and ${\left\{Y_{t}\right\}}_{t=1}^{n_{2}}$ is not a necessary assumption in our method. We only require the pair sequence $\left\{\left(X_{t},Y_{t}\right)\right\}$ is weakly dependent. Therefore, our method can be applied effectively to detect change points in high-dimensional time series. Further details on this application can be found in Section 4.

The rest of this paper is organized as follows. Section 2 introduces the test statistic and the blockwise bootstrap method. The convergence rates of Gaussian approximations for high-dimensional α-mixing sequence and the theoretical properties of the proposed test can be found in Section 3. In Section 4, an application to change point detection for high-dimensional time series is presented. The selection method for tuning parameter and a simulation study to investigate the numerical performance of the test are displayed in Section 5. We apply the proposed method to the opening price data from multiple stocks in Section 6. Section 7 provides discussions on the results and outlines our future work. The proofs of the main results in Section 3 are detailed in the Appendix A, Appendix B, Appendix C and Appendix D.

**Notation:**  For any positive integer p≥1, we write [p]={1,…,p}. We use ${\left|a\right|}_{\infty }=\max_{j\in [p]}\left|a_{j}\right|$ to denote the $\ell_{\infty }$-norm of the *p*-dimensional vector a. Let ⌊x⌋ and ⌈x⌉ represent the greatest integer less than or equal to *x* and the smallest integer greater than or equal to *x*, respectively. For two sequences of positive numbers $\left\{a_{n}\right\}$ and $\left\{b_{n}\right\}$, we write $a_{n}≲b_{n}$ or $b_{n}≳a_{n}$ if ${lim sup}_{n\to \infty }a_{n}/b_{n}⩽c_{0}$ for some positive constant $c_{0}$. Let $a_{n}≍b_{n}$ if $a_{n}≲b_{n}$ and $b_{n}≲a_{n}$ hold simultaneously. Denote $0_{p}={\left(0,\ldots ,0\right)}^{T}\in R^{p}$. For any m×m matrix $A={\left(a_{ij}\right)}_{m\times m}$, let ${\left|A\right|}_{\infty }=\max_{i,j\in [m]}\left|a_{ij}\right|$ and ${∥A∥}_{2}$ be the spectral norm of A. Additionally, denote $\lambda_{\min}\left(A\right)$ as the smallest eigenvalue of A. Let 1(·) be the indicator function. For any x,y∈R, denote x∨y=max{x,y} and x∧y=min{x,y}. Given γ>0, we define the function $\psi_{\gamma }\left(x\right):=\exp\left(x^{\gamma }\right)-1$ for any x>0. For a real-valued random variable ξ, we define ${∥\xi ∥}_{\psi_{\gamma }}:=\inf[\lambda >0:E\{\psi_{\gamma }\left(|\xi |/\lambda )\}\leq 1\right]$. Throughout the paper, we use c,C∈(0,∞) to denote two generic finite constants that do not depend on $\left(n_{1},n_{2},p\right)$, and may be different in different uses.

## 2. Methodology

### 2.1. Test Statistic and Its Gaussian Analog

Consider two weakly stationary time series $\left\{X_{t},t\in Z\right\}$ and $\left\{Y_{t},t\in Z\right\}$ with $X_{t}={\left(X_{t,1},\ldots ,X_{t,p}\right)}^{T}$ and $Y_{t}={\left(Y_{t,1},\ldots ,Y_{t,p}\right)}^{T}$. Let $\mu_{X}=E\left(X_{t}\right)$ and $\mu_{Y}=E\left(Y_{t}\right)$. The primary focus is on testing equality of mean vectors of the two populations:

$$\begin{array}{c} H_{0}:\mu_{X}=\mu_{Y}\;\mathrm{versus}\;H_{1}:\mu_{X}\neq \mu_{Y}. \end{array}$$

Given the observations ${\left\{X_{t}\right\}}_{t=1}^{n_{1}}$ and ${\left\{Y_{t}\right\}}_{t=1}^{n_{2}}$, the estimations of $\mu_{X}$ and $\mu_{Y}$ are, respectively, ${\hat{\mu }}_{X}={n_{1}}^{-1}\sum_{t=1}^{n_{1}}X_{t}$ and ${\hat{\mu }}_{Y}={n_{2}}^{-1}\sum_{t=1}^{n_{2}}Y_{t}$. In this paper, we assume $n_{1}≍n_{2}≍n$. It is natural to consider the $\ell_{\infty }$-type test statistic $T_{n}={\left(n_{1}n_{2}\right)}^{1/2}{\left(n_{1}+n_{2}\right)}^{-1/2}{\left|{\hat{\mu }}_{X}-{\hat{\mu }}_{Y}\right|}_{\infty }$. Write $\tilde{n}=\max\left\{n_{1},n_{2}\right\}$. Define two new sequences ${\left\{{\tilde{X}}_{t}\right\}}_{t=1}^{\tilde{n}}$ and ${\left\{{\tilde{Y}}_{t}\right\}}_{t=1}^{\tilde{n}}$ with

$$\begin{array}{c} {\tilde{X}}_{t}=X_{t\wedge n_{1}}1\left(1\leq t\leq n_{1}\right)\;\;\mathrm{and}\;\;{\tilde{Y}}_{t}=Y_{t\wedge n_{2}}1\left(1\leq t\leq n_{2}\right). \end{array}$$

For each $t\in [\tilde{n}]$, let

$$Z_{t}=\sqrt{\frac{n_{2}\tilde{n}}{n_{1}\left(n_{1}+n_{2}\right)}}{\tilde{X}}_{t}-\sqrt{\frac{n_{1}\tilde{n}}{n_{2}\left(n_{1}+n_{2}\right)}}{\tilde{Y}}_{t}.$$

Then, $T_{n}$ can be rewritten as

(2) $$\begin{array}{c} T_{n}=|\frac{1}{\sqrt{\tilde{n}}}\sum_{t=1}^{\tilde{n}}Z_{t}{|}_{\infty }. \end{array}$$

We reject the null hypothesis $H_{0}$ if $T_{n}>{\mathrm{cv}}_{\alpha }$, where ${\mathrm{cv}}_{\alpha }$ represents the critical value at the significance level α∈(0,1). Determining ${\mathrm{cv}}_{\alpha }$ involves deriving the distribution of $T_{n}$ under $H_{0}$. However, due to the divergence of *p* in a high-dimensional scenario, obtaining the distribution of $T_{n}$ is challenging. To address this challenge, we employ the Gaussian approximation theorem [15,16]. We seek a Gaussian analog, denoted as $T_{n}^{G}$, satisfying the property that the Kolmogorov distance between $T_{n}$ and $T_{n}^{G}$ converges to zero under $H_{0}$. Then, we can replace ${\mathrm{cv}}_{\alpha }$ by ${\mathrm{cv}}_{\alpha }^{G}:=\inf\left\{x>0:P\left(T_{n}^{G}>x\right)\leq \alpha \right\}$. Define a *p*-dimensional Gaussian vector

(3) $$\begin{array}{c} G∼N\left(0_{p},\Xi_{\tilde{n}}\right)\;\;\mathrm{with}\;\;\Xi_{\tilde{n}}=\mathrm{var}\left(\frac{1}{\sqrt{\tilde{n}}}\sum_{t=1}^{\tilde{n}}Z_{t}\right). \end{array}$$

We then define the Gaussian analogue of $T_{n}$ as

$$\begin{array}{c} T_{n}^{G}={\left|G\right|}_{\infty }. \end{array}$$

Proposition 1 below demonstrates that the null distribution of $T_{n}$ can be effectively approximated by the distribution of $T_{n}^{G}$.

### 2.2. Blockwise Bootstrap

Note that the long-run covariance matrix $\Xi_{\tilde{n}}$ specified in (3) is typically unknown. As a result, determining ${\mathrm{cv}}_{\alpha }^{G}$ through the distribution of $T_{n}^{G}$ becomes challenging. To address this challenge, we introduce a parametric bootstrap estimator for $T_{n}$ using the blockwise bootstrap method [18].

For some positive constant ϑ∈[1/2,1), let $S≍{\tilde{n}}^{1-\vartheta }$ and $B=\lceil \tilde{n}/S\rceil$ be the size of each block and the number of blocks, respectively. Denote $I_{b}=\left\{\left(b-1\right)S+1,\ldots ,bS\right\}$ for b∈[B−1] and $I_{B}=\left\{\left(B-1\right)S+1,\ldots ,\tilde{n}\right\}$. Let ${\left\{\varrho_{b}\right\}}_{b=1}^{B}$ be the sequence of i.i.d. standard normal random variables and $\varrho^{\prime }=\left(\varrho_{1}^{\prime },\ldots ,\varrho_{\tilde{n}}^{\prime }\right)$, where $\varrho_{t}^{\prime }=\varrho_{b}$ if $t\in I_{b}$. Define the bootstrap estimator of $T_{n}$ as

$$\begin{array}{c} {\hat{T}}_{n}^{G}=|\frac{1}{\sqrt{\tilde{n}}}\sum_{t=1}^{\tilde{n}}\left(Z_{t}-\bar{Z}\right)\varrho_{t}^{\prime }{|}_{\infty }, \end{array}$$

where $\bar{Z}={\tilde{n}}^{-1}\sum_{t=1}^{\tilde{n}}Z_{t}$. Based on this estimator, we define the estimated critical value ${\hat{\mathrm{cv}}}_{\alpha }$ as

(4) $$\begin{array}{cc} {\hat{\mathrm{cv}}}_{\alpha }:= & \;\inf\{x>0:P\left({\hat{T}}_{n}^{G}>x\;|\;E\right)\leq \alpha \}, \end{array}$$

where $E=\{X_{1},\ldots ,X_{n_{1}},Y_{1},\ldots ,Y_{n_{2}}\}$. Then, we reject the null hypothesis $H_{0}$ if $T_{n}>{\hat{\mathrm{cv}}}_{\alpha }$. The procedure for selecting the parameter ϑ (or block size *S*) is detailed in Section 5.1. In practice, we obtain ${\hat{\mathrm{cv}}}_{\alpha }$ through the following bootstrap procedure: Generate *K* independent sequences ${\left\{\varrho_{(1),t}^{\prime }\right\}}_{t=1}^{\tilde{n}},\ldots ,{\left\{\varrho_{(K),t}^{\prime }\right\}}_{t=1}^{\tilde{n}}$, with each ${\left\{\varrho_{(k),t}^{\prime }\right\}}_{t=1}^{\tilde{n}}$ generated as ${\left\{\varrho_{t}^{\prime }\right\}}_{t=1}^{\tilde{n}}$. For each k∈[K], calculate ${\hat{T}}_{(k),n}^{G}$ with ${\left\{\varrho_{(k),t}^{\prime }\right\}}_{t=1}^{\tilde{n}}$. Then, ${\hat{\mathrm{cv}}}_{\alpha }$ is the (1−α)K-th largest value among $\left\{{\hat{T}}_{(1),n}^{G},\ldots ,{\hat{T}}_{(K),n}^{G}\right\}$. Here, *K* is the number of bootstrap replications.

## 3. Theoretical Results

We employ the concept of ‘α-mixing’ to characterize the serial dependence of $\left\{\left(X_{t},Y_{t}\right)\right\}$, with the α-mixing coefficient at lag κ defined as

(5) $$\begin{array}{c} \alpha \left(\kappa \right):=\underset{r}{\sup}\underset{A\in F_{-\infty }^{r},B\in F_{r+\kappa }^{\infty }}{\sup}\left|P\left(AB\right)-P\left(A\right)P\left(B\right)\right|, \end{array}$$

where $F_{-\infty }^{r}$ and $F_{r+\kappa }^{\infty }$ are the σ-fields generated by $\left\{\left(X_{t},Y_{t}\right):t\leq r\right\}$ and $\left\{\left(X_{t},Y_{t}\right):t\geq r+\kappa \right\}$, respectively. We call the sequence $\left\{\left(X_{t},Y_{t}\right)\right\}$ is α-mixing if α(κ)→0 as κ→∞.

### 3.1. Gaussian Approximation for High-Dimensional α-Mixing Sequence

To show that the Kolmogorov distance between $T_{n}$ and $T_{n}^{G}$ converges to zero under various divergence rates of *p*, we need the following central limit theorems for high-dimensional α-mixing sequence.

**Theorem** **1.** *Let ${\left\{\xi_{t}\right\}}_{t=1}^{n}$ be an α-mixing sequence of p-dimensional centered random vectors and ${\left\{\alpha \left(\kappa \right)\right\}}_{\kappa \geq 1}$ denote the α-mixing coefficients of $\left\{\xi_{t}\right\}$, defined in the same manner as (5). Write $S_{n}={\left(S_{n,1},\ldots ,S_{n,p}\right)}^{T}=n^{-1/2}\sum_{t=1}^{n}\xi_{t}$ and $W={\left(W_{1},\ldots ,W_{p}\right)}^{T}∼N\left(0_{p},\Sigma_{n}\right)$ with $\Sigma_{n}=E\left(S_{n}S_{n}^{T}\right)$. Define*

$$\rho_{n}=\underset{x\in R}{\sup}\left|P(\right|S_{n}{|}_{\infty }\leq {x)-P(|W|}_{\infty }\leq x)|.$$

*(i)*  If $\max_{t\in [n]}\max_{j\in [p]}E(|\xi_{t,j}{|}^{m})\leq C_{1}^{*}$, $\alpha \left(\kappa \right)\leq C_{2}^{*}\kappa^{-\tau }$ and $\lambda_{\min}\left(\Sigma_{n}\right)\geq C_{3}^{*}$ for some m>3, τ>max{2m/(m−3),3} and constants $C_{1}^{*},C_{2}^{*},C_{3}^{*}>0$, we have
$$\rho_{n}≲\frac{p^{1/2}{\left(\log p\right)}^{1/4}}{n^{\tilde{\tau }}}$$
  *provided that $p=o(n^{2\tilde{\tau }})$, where $\tilde{\tau }=\tau /\left(11\tau +12\right)$.*
*(ii)*  If $\max_{t\in [n]}\max_{j\in [p]}{∥\xi_{t,j}∥}_{\psi_{\gamma_{1}}}\leq M_{n}$, $\alpha \left(\kappa \right)\leq C_{1}^{**}\exp\left(-C_{2}^{**}\kappa^{\gamma_{2}}\right)$ and $\min_{j\in [p]}{\left(\Sigma_{n}\right)}_{j,j}\geq C_{3}^{**}$ for some $M_{n}\geq 1$, $\gamma_{1}\in \left(0,2\right]$, $\gamma_{2}>0$ and constants $C_{1}^{**},C_{2}^{**},C_{3}^{**}>0$, we have
$$\rho_{n}≲\frac{M_{n}{\left\{\log\left(pn\right)\right\}}^{\max\{\left(2\gamma_{2}+1\right)/2\gamma_{2},3/2\}}}{n^{1/6}}$$
  *provided that ${\left\{\log\left(pn\right)\right\}}^{3}=o\left\{n^{\gamma_{1}\gamma_{2}/\left(2\gamma_{1}+2\gamma_{2}-\gamma_{1}\gamma_{2}\right)}\right\}$ and $M_{n}^{2}{\left\{\log\left(pn\right)\right\}}^{1/\gamma_{2}}=o\left(n^{1/3}\right)$.*

**Remark** **1.** *In scenarios where the dimension p diverges polynomially with respect to n, Theorem 1(i) represents a novel contribution to the existing literature. Moreover, if τ→∞(i.e., α(κ)≲exp(−Cκ) for some constant C>0), we have $\tilde{\tau }\to 1/11$, and thus $\rho_{n}=o\left(1\right)$ if $p{\left(\log p\right)}^{1/2}=o\left(n^{2/11}\right)$. Compared with Theorem 1 in [14], which provides a Gaussian approximation result when p diverges exponentially with respect to n, Theorem 1(ii) has three improvements. Firstly, all conditions of Theorem 1(ii) are equivalent to those in Theorem 1 of [14], with the exception that we permit $\gamma_{1}\in \left(0,1\right)$, thereby offering a weaker assumption that is more broadly applicable. Secondly, the convergence rate dependent on n via $n^{-1/6}$ in Theorem 1(ii) outperforms the rate of $n^{-1/9}$ demonstrated in Theorem 1 of [14]. Note that the convergence rate in Theorem 1 of [14] can be rewritten as*

$${\left[\frac{M_{n}{\left\{\log\left(pn\right)\right\}}^{\left(2\gamma_{2}+1\right)/2\gamma_{2}}}{n^{1/6}}\right]}^{2/3}+\frac{M_{n}{\left\{\log\left(pn\right)\right\}}^{7/6}}{n^{1/9}}.$$

*To ensure $\rho_{n}=o\left(1\right)$, in our result, it is necessary to allow $M_{n}^{6}{\left\{\log\left(pn\right)\right\}}^{\left(6\gamma_{2}+3\right)/\gamma_{2}}=o\left(n\right)$ when $\gamma_{2}\leq 2/3$ and $M_{n}^{6}{\left\{\log\left(pn\right)\right\}}^{\max\{\left(6\gamma_{2}+3\right)/\gamma_{2},9\}}=o\left(n\right)$ when $\gamma_{2}>2/3$, respectively. Comparatively, the basic requirements under Theorem 1 of [14] are $M_{n}^{6}{\left\{\log\left(pn\right)\right\}}^{\left(6\gamma_{2}+3\right)/\gamma_{2}}=o\left(n\right)$ when $\gamma_{2}\leq 2/3$ and $M_{n}^{9}{\left\{\log\left(pn\right)\right\}}^{21/2}=o\left(n\right)$ when $\gamma_{2}>2/3$, respectively. Due to $\left(6\gamma_{2}+3\right)/\gamma_{2}<21/2$ when $\gamma_{2}>2/3$, our result permits a larger or equal divergence rate of p compared with Theorem 1 in [14].*

### 3.2. Theoretical Properties

In order to derive the theoretical properties of $T_{n}$, the following regular assumptions are needed.

**Assumption** **1.**

*(i)*  *For some m>4, there exists a constant $C_{1}>0$ s.t. $\max_{t\in [\tilde{n}]}\max_{j\in [p]}E(|Z_{t,j}{|}^{m})\leq C_{1}$.*
*(ii)*  *There exists a constant $C_{2}>0$ s.t. $\alpha \left(\kappa \right)\leq C_{2}\kappa^{-\tau }$ for some τ>3m/(m−4).*
*(iii)*  *There exists a constant $C_{3}>0$ s.t. $\lambda_{\min}\left(\Xi_{\tilde{n}}\right)\geq C_{3}$.*

**Assumption** **2.**

*(i)*  *There exists a constant $C_{1}^{\prime }>0$ s.t. $\max_{t\in [\tilde{n}]}\max_{j\in [p]}{∥Z_{t,j}∥}_{\psi_{2}}\leq C_{1}^{\prime }$.*
*(ii)*  *There exist two constants $C_{2}^{\prime },C_{3}^{\prime }>0$ s.t. $\alpha \left(\kappa \right)\leq C_{2}^{\prime }\exp\left(-C_{3}^{\prime }\kappa \right)$.*
*(iii)*  *There exists a constant $C_{4}^{\prime }>0$ s.t. $\min_{j\in [p]}{\left(\Xi_{\tilde{n}}\right)}_{j,j}\geq C_{4}^{\prime }$.*

**Remark** **2.** *The two mild Assumptions, 1 and 2, delineate the necessary assumptions for $\left\{\left(X_{t},Y_{t}\right)\right\}$ to facilitate the development of Gaussian approximation theories for the dimension p divergence, characterized by polynomial and exponential rates relative to the sample size n, respectively. Assumptions 1(i) and 1(ii) are common assumptions in multivariate time series analysis. Due to $n_{1}≍n_{2}≍n$, if $\max_{t\in \left[n_{1}\right],j\in \left[p\right]}E(|X_{t,j}{|}^{m})\leq C$ and $\max_{t\in \left[n_{2}\right],j\in \left[p\right]}E(|Y_{t,j}{|}^{m})\leq C$, then Assumption 1(i) holds, as verified by the triangle inequality. Additionally, Assumption 1(iii) necessitates the strong nondegeneracy of $\Xi_{\tilde{n}}$, a requirement commonly assumed in Gaussian approximation theories (see refs. [21,22], among others). Note that Assumption 2(iii) is implied by Assumption 1(iii). The latter assumption only necessitates the nondegeneracy of $\min_{j\in [p]}\mathrm{var}\left({\tilde{n}}^{-1/2}\sum_{t=1}^{\tilde{n}}Z_{t,j}\right)$. We can modify Assumption 2(i) to $\max_{t\in [\tilde{n}]}\max_{j\in [p]}{∥Z_{t,j}∥}_{\psi_{\gamma }}\leq C$ for any γ∈(0,2], a standard assumption in the literature on ultra-high-dimensional data analysis. This assumption ensures subexponential upper bounds for the tail probabilities of the statistics in question when p≫n, as discussed in [23,24]. The requirement of sub-Gaussian properties in Assumption 2(i) is made for the sake of simplicity. If $\left\{X_{t}\right\}$ and $\left\{Y_{t}\right\}$ share the same tail probability, Assumption 2(i) is satisfied automatically. Assumption 2(ii) necessitates that the α-mixing coefficients decay at an exponential rate.*

Write $\Delta_{n}:=\max\left\{n_{1},n_{2}\right\}-\min\left\{n_{1},n_{2}\right\}$. Define two cases with respect to the distinct divergence rates of *p* as

- Case1: ${\left\{X_{t}\right\}}_{t=1}^{n_{1}}$ and ${\left\{Y_{t}\right\}}_{t=1}^{n_{2}}$ satisfy Assumption 1, and the dimension *p* satisfies $p^{2}\log p=o\left\{n^{4\tau /(11\tau +12)}\right\}$ and $\Delta_{n}^{2}\log p=o\left(n\right)$;
- Case2: ${\left\{X_{t}\right\}}_{t=1}^{n_{1}}$ and ${\left\{Y_{t}\right\}}_{t=1}^{n_{2}}$ satisfy Assumption 2, and the dimension *p* satisfies $\log\left(pn\right)=o\left(n^{1/9}\right)$ and $\Delta_{n}^{2}\log p=o\left(n\right)$.

Note that $\Delta_{n}^{2}\log p=o\left(n\right)$ mandates the maximum difference between the two sample sizes. Proposition 1 below demonstrates that, under the aforementioned cases and $H_{0}$, the Kolmogorov distance between $T_{n}$ and $T_{n}^{G}$ converges to zero as the sample size approaches infinity. Proposition 1 can be directly derived from Theorem 1. Note that, in the scenario where the dimension *p* diverges in a polynomial rate with respect to *n*, obtaining Proposition 1 requires only m>3 and τ>max{2m/(m−3),3}, an assumption weaker than Assumption 1. The more stringent restrictions m>4 and τ>3m/(m−4) in Assumption 1 are imposed to establish the results presented in Theorems 2 and 3.

**Proposition** **1.** *In either* Case1 *or* Case2, *it holds under the null hypothesis $H_{0}$ that*

$$\begin{array}{c} \underset{x\in R}{\sup}\left|P\left(T_{n}\leq x\right)-P\left(T_{n}^{G}\leq x\right)\right|=o\left(1\right). \end{array}$$

According to Proposition 1, the critical value ${\mathrm{cv}}_{\alpha }$ can be substituted with ${\mathrm{cv}}_{\alpha }^{G}$. However, in practical scenarios, the long-run covariance $\Xi_{\tilde{n}}$ defined in (3) is typically unknown. This implies that obtaining ${\mathrm{cv}}_{\alpha }^{G}$ directly from the distribution of $T_{n}^{G}$ is not feasible. We introduce a bootstrap method for obtaining the estimator ${\hat{\mathrm{cv}}}_{\alpha }$ defined in (4). In situations where the dimension *p* diverges at a polynomial rate relative to the sample size *n*, we require an additional Assumption 3 to ensure that ${\hat{\mathrm{cv}}}_{\alpha }$ serves as a reliable estimator for ${\mathrm{cv}}_{\alpha }$. Assumption 3 places restrictions on the cumulant function, a commonly assumed criterion in time series analysis. Refer to [25,26] for examples of such assumptions in the literature.

**Assumption** **3.** *For each i,j∈[p], define ${\mathrm{cum}}_{i,j}\left(h,t,s\right)=\mathrm{cov}\left({\overset{˚}{Z}}_{0,i}{\overset{˚}{Z}}_{h,j},{\overset{˚}{Z}}_{t,i}{\overset{˚}{Z}}_{s,j}\right)-\gamma_{t,i,i}\gamma_{s-h,j,j}-\gamma_{s,i,j}\gamma_{t-h,j,i}$, where $\gamma_{h,i,j}=\mathrm{cov}\left(Z_{0,i},Z_{h,j}\right)$ and ${\overset{˚}{Z}}_{t,j}=Z_{t,j}-E\left(Z_{t,j}\right)$. There exists a constant $C_{4}>0$ s.t.*

$$\max_{i,j\in [p]}\sum_{h=-\infty }^{\infty }\sum_{t=-\infty }^{\infty }\sum_{s=-\infty }^{\infty }\left|{\mathrm{cum}}_{i,j}\left(h,t,s\right)\right|<C_{4}.$$

Similar to Case1 and Case2, we consider two cases corresponding to different divergence rates of the dimension *p*, as outlined below:

- Case3: ${\left\{X_{t}\right\}}_{t=1}^{n_{1}}$ and ${\left\{Y_{t}\right\}}_{t=1}^{n_{2}}$ satisfy Assumptions 1 and 3.
- Case4: ${\left\{X_{t}\right\}}_{t=1}^{n_{1}}$ and ${\left\{Y_{t}\right\}}_{t=1}^{n_{2}}$ satisfy Assumption 2.

**Theorem** **2.** *In either* Case3 *with $p\log p=o[n^{\min\{(1-\vartheta )/4,2\tau /(11\tau +12)\}}]$ and $\Delta_{n}^{2}\log p=o\left(n\right)$, or* Case4 *with $\log\left(pn\right)=o\left[n^{\min\{(1-\vartheta )/2,\vartheta /7,1/9\}}\right]$ and $\Delta_{n}^{2}\log p=o\left(n\right)$, it holds under $H_{0}$ that $\sup_{x\in R}\left|P\left(T_{n}\leq x\right)-P\left({\hat{T}}_{n}^{G}\leq x\;|\;E\right)\right|=o_{p}\left(1\right)$. Moreover, it holds under $H_{0}$ that*

$$P(T_{n}>{\hat{\mathrm{cv}}}_{\alpha })\to \alpha \;\;as\;\;n\to \infty .$$

**Theorem** **3.** *In either* Case3 *with $p=o\{n^{(1-\vartheta )/4}\}$ or* Case4 *with $\log\left(pn\right)=o\left[n^{\min\{\vartheta /3,(1-\vartheta )/2\}}\right]$, if $\max_{j\in [p]}\left|\mu_{X,j}-\mu_{Y,j}\right|\gg n^{-1/2}{\left(\log p\right)}^{1/2}$, it holds that*

$$P(T_{n}>{\hat{\mathrm{cv}}}_{\alpha })\to 1\;\;as\;\;n\to \infty .$$

**Remark** **3.** *The different requirements for the divergence rates of p follow from the fact that we do not rely on the Gaussian approximation and comparison results under certain alternative hypotheses. By Theorem 2 and Theorem 3, the optimal selections for ϑ are 1/2 and 7/9 in Case3 and Case4, respectively. This implies that $\lim_{n\to \infty }P_{H_{0}}\left(T_{n}>{\hat{\mathrm{cv}}}_{\alpha }\right)=\alpha$ holds with $p\log p=o(n^{1/8})$ in Case3 and $\log\left(pn\right)=o\left(n^{1/9}\right)$ in Case4. Under certain alternative hypotheses, $\lim_{n\to \infty }P_{H_{1}}\left(T_{n}>{\hat{\mathrm{cv}}}_{\alpha }\right)=1$ holds with $p=o(n^{1/8})$ in Case3 and $\log\left(pn\right)=o\left(n^{1/9}\right)$ in Case4.*

## 4. Application: Change Point Detection

In this section, we elaborate that our two-sample testing procedure can be regarded as a novel method for detecting change points for high-dimensional time series. To illustrate, we provide a notation for the detection of a single change point, with the understanding that it can be easily extended to the multiple change points case.

Consider a *p*-dimensional time series ${\left\{X_{t}\right\}}_{t=1}^{n}$. Let $\mu_{t}=E\left(X_{t}\right)$. Consider the following hypothesis testing problem:

$$H_{0}^{\prime }:\mu_{1}=\cdots =\mu_{n}\;\;\;\mathrm{versus}\;\;\;H_{1}^{\prime }:\mu_{1}=\cdots =\mu_{\tau_{0}-1}\neq \mu_{\tau_{0}}=\cdots =\mu_{n}.$$

Here, $\tau_{0}$ is the unknown change point. Let *w* be a positive integer such that $w<\min\{\tau_{0},n-\tau_{0}\}$. We define ${\bar{\mu }}^{t}=w^{-1}\sum_{l=t-w/2+1}^{t+w/2}\mu_{l}$, ${\bar{\mu }}^{\left(1\right)}=w^{-1}\sum_{l=1}^{w}\mu_{l}$ and ${\bar{\mu }}^{\left(2\right)}=w^{-1}\sum_{l=n-w+1}^{n}\mu_{l}$. Then for each t∈[3w/2,n−3w/2], define $\Delta^{t,(1)}={\bar{\mu }}^{t}-{\bar{\mu }}^{\left(1\right)}$ and $\Delta^{t,(2)}={\bar{\mu }}^{t}-{\bar{\mu }}^{\left(2\right)}$. Thus,

$$\begin{array}{c} \Delta^{t,(1)}=\left\{\begin{array}{cc} 0_{p} & \;,\;\;\;\mathrm{if}\;\;3w/2\leq t\leq \tau_{0}-w/2,\\ \left({\bar{\mu }}^{\left(2\right)}-{\bar{\mu }}^{\left(1\right)}\right)\frac{t+w/2-\tau_{0}}{w} & \;,\;\;\;\mathrm{if}\;\;\tau_{0}-w/2<t\leq \tau_{0}+w/2,\\ {\bar{\mu }}^{\left(2\right)}-{\bar{\mu }}^{\left(1\right)} & \;,\;\;\;\mathrm{if}\;\;\tau_{0}+w/2<t\leq n-3w/2, \end{array}\right.\\ \Delta^{t,(2)}=\left\{\begin{array}{cc} {\bar{\mu }}^{\left(1\right)}-{\bar{\mu }}^{\left(2\right)} & \;,\;\;\;\mathrm{if}\;\;3w/2\leq t\leq \tau_{0}-w/2,\\ \left({\bar{\mu }}^{\left(1\right)}-{\bar{\mu }}^{\left(2\right)}\right)\frac{-t+w/2+\tau_{0}}{w} & \;,\;\;\;\mathrm{if}\;\;\tau_{0}-w/2<t\leq \tau_{0}+w/2,\\ 0_{p} & \;,\;\;\;\mathrm{if}\;\;\tau_{0}+w/2<t\leq n-3w/2. \end{array}\right. \end{array}$$

Assume $|{\bar{\mu }}^{\left(1\right)}-{\bar{\mu }}^{\left(2\right)}{|}_{\infty }=O\left(1\right)$, which represents the sparse signals case. Define $t_{1}\left(\varepsilon^{t,(1)}\right)=\min\{t\in \left[3w/2,n-3w/2\right]:|\Delta^{t,(1)}\left|>\varepsilon^{t,(1)}\right\}$ and $t_{2}\left(\varepsilon^{t,(2)}\right)=\max\{t\in \left[3w/2,n-3w/2\right]:|\Delta^{t,(2)}\left|>\varepsilon^{t,(2)}\right\}$ with two well-defined thresholds $\varepsilon^{t,(1)},\varepsilon^{t,(2)}\geq 0$. Due to the symmetry of $|\Delta^{t,(1)}|$ and $|\Delta^{t,(2)}|$, it holds under $H_{1}^{\prime }$ that

$$\begin{array}{c} \tau_{0}=\frac{t_{1}\left(\varepsilon^{t,(1)}\right)+t_{2}\left(\varepsilon^{t,(2)}\right)}{2}. \end{array}$$

The sample estimators of ${\bar{\mu }}^{t}$, ${\bar{\mu }}^{\left(1\right)}$ and ${\bar{\mu }}^{\left(2\right)}$ are, respectively, ${\hat{\bar{\mu }}}^{t}=w^{-1}\sum_{l=t-w/2+1}^{t+w/2}X_{l}$, ${\hat{\bar{\mu }}}^{\left(1\right)}=w^{-1}\sum_{l=1}^{w}X_{l}$ and ${\hat{\bar{\mu }}}^{\left(2\right)}=w^{-1}\sum_{l=n-w+1}^{n}X_{l}$. Based on the method proposed in Section 2, with $n_{1}=n_{2}=w$, we define the following two test statistics:

$$T_{w}^{t,(1)}=\sqrt{w}|{\hat{\bar{\mu }}}^{t}-{\hat{\bar{\mu }}}^{\left(1\right)}{|}_{\infty }\;\;\mathrm{and}\;\;T_{w}^{t,(2)}=\sqrt{w}{\left|{\hat{\bar{\mu }}}^{t}-{\hat{\bar{\mu }}}^{\left(2\right)}\right|}_{\infty }.$$

Given a significance level α>0, we choose $\varepsilon^{t,(1)}={\mathrm{cv}}_{1\alpha }^{t}$ and $\varepsilon^{t,(2)}={\mathrm{cv}}_{2\alpha }^{t}$, where ${\mathrm{cv}}_{1\alpha }^{t}$ and ${\mathrm{cv}}_{2\alpha }^{t}$ are, respectively, the (1−α)-quantiles of the distributions of $T_{w}^{t,(1)}$ and $T_{w}^{t,(2)}$. The estimated critical values ${\hat{\mathrm{cv}}}_{1\alpha }^{t}$ and ${\hat{\mathrm{cv}}}_{2\alpha }^{t}$ can be obtained by (4). Thus, ${\hat{t}}_{1}=\min\left\{t\in \left[3w/2,n-3w/2\right]:T_{w}^{t,(1)}>{\hat{\mathrm{cv}}}_{1\alpha }^{t}\right\}$ and ${\hat{t}}_{2}=\max\left\{t\in \left[3w/2,n-3w/2\right]:T_{w}^{t,(2)}>{\hat{\mathrm{cv}}}_{2\alpha }^{t}\right\}$. Hence, the estimator of $\tau_{0}$ is given by

$$\begin{array}{c} {\hat{\tau }}_{0}=\frac{{\hat{t}}_{1}+{\hat{t}}_{2}}{2}. \end{array}$$

We utilize $T_{w}^{t,(1)}$ as an illustrative example to elucidate the applicability of our proposed method. Let *w* be an even integer. For any t∈[5w/2,n−3w/2], we have $T_{w}^{t,(1)}={\left|w^{-1/2}\sum_{l=1}^{w}\left(X_{t-w/2+l}-X_{l}\right)\right|}_{\infty }$, where the sequence ${\left\{X_{t-w/2+l}-X_{l}\right\}}_{l=1}^{w}$ possesses the same weakly dependence properties and similar moment/tail conditions as ${\left\{X_{l}\right\}}_{l=1}^{n}$. For t∈[3w/2,5w/2−1], let ${\left\{{\tilde{X}}_{l}\right\}}_{l=1}^{t-w/2}$ be defined as ${\tilde{X}}_{l}=X_{l}$ when l∈[1,w] and ${\tilde{X}}_{l}=0_{p}$ when l∈[w+1,t−w/2]. Additionally, define ${\left\{{\tilde{Y}}_{l}\right\}}_{l=t-w/2+1}^{2t-w}$ as ${\tilde{Y}}_{l}=X_{l}$ when l∈[t−w/2+1,t+w/2] and ${\tilde{Y}}_{l}=0_{p}$ when l∈[t+w/2+1,2t−w]. Then, $T_{w}^{t,(1)}$ can be expressed as $|w^{-1/2}\sum_{l=1}^{t/2-w/4}\left\{\left({\tilde{Y}}_{t-w/2+l}-{\tilde{X}}_{l}\right)+\left({\tilde{Y}}_{2t-w+1-l}-{\tilde{X}}_{t-w/2+1-l}\right)\right\}{|}_{\infty }$, and ${\left\{\left({\tilde{Y}}_{t-w/2+l}-{\tilde{X}}_{l}\right)+\left({\tilde{Y}}_{2t-w+1-l}-{\tilde{X}}_{t-w/2+1-l}\right)\right\}}_{l=1}^{t/2-w/4}$ shares the same weakly dependence properties and similar moment/tail conditions as ${\left\{X_{l}\right\}}_{l=1}^{n}$. Hence, our method can be applied to change point detection.

The selections of *w* and α are crucial in this method. We will elaborate on the specific choices for them in future works.

## 5. Simulation Study

### 5.1. Tuning Parameter Selection

Given the observations ${\left\{X_{t}\right\}}_{t=1}^{n_{1}}$ and ${\left\{Y_{t}\right\}}_{t=1}^{n_{2}}$, we use the minimum volatility (MV) method proposed in [27] to select the block size *S*.

When the data are independent, by the multiplier bootstrap method described in [28], we set $B=\tilde{n}$ (thus S=1). In this case,

$$\begin{array}{cc} {\hat{\Xi }}_{\tilde{n}}= & \;\mathrm{var}\left(\frac{1}{\sqrt{\tilde{n}}}\sum_{t=1}^{\tilde{n}}\left(Z_{t}-\bar{Z}\right)\varrho_{t}^{\prime }\;|\;Z_{1},\ldots ,Z_{\tilde{n}}\right)\\ = & \;\frac{1}{\tilde{n}}\sum_{b=1}^{B}\left\{\left(\sum_{t\in I_{b}}\left(Z_{t}-\bar{Z}\right)\right){\left(\sum_{t\in I_{b}}\left(Z_{t}-\bar{Z}\right)\right)}^{T}\right\}=\frac{1}{\tilde{n}}\sum_{t=1}^{\tilde{n}}\left(Z_{t}-\bar{Z}\right){\left(Z_{t}-\bar{Z}\right)}^{T} \end{array}$$

proves to be a reliable estimator of $\Xi_{\tilde{n}}$ introduced in Section 3. When the data are weakly dependent (and thus nearly independent), we expect a small value for *S* and a large value for *B*. Therefore, we recommend exploring a narrow range of *S*, such as S∈{1,…,m}, where *m* is a moderate integer. In our theoretical proof, the quality of the bootstrap approximation depends on how well the ${\hat{\Xi }}_{\tilde{n}}$ approximates the covariance $\Xi_{\tilde{n}}$. The idea behind the MV method is that the conditional covariance ${\hat{\Xi }}_{\tilde{n}}$ should exhibit stable behavior as a function of *S* within an appropriate range. For more comprehensive discussions on the MV method and its applications in time series analysis, we refer readers to [27,29]. For a moderately sized integer *m*, let $S_{1}<S_{2}<\cdots <S_{m}$ be a sequence of equally spaced candidate block sizes, and $S_{0}=2S_{1}-S_{2}$, $S_{m+1}=2S_{m}-S_{m-1}$. For each i∈{0,…,m+1}, let

$$Y_{j}^{i}=\sum_{b=1}^{B(S_{i})}{\left\{\sum_{t\in I_{b}}\left(Z_{t,j}-{\bar{Z}}_{j}\right)\right\}}^{2},$$

where j∈[p] and $B\left(S\right)=\left\lceil \tilde{n}/S\right\rceil$. Then for each i∈{1,…,m}, we compute

$$Y^{i}=\sum_{j=1}^{p}\mathrm{sd}\left({\left\{Y_{j}^{l}\right\}}_{l=i-1}^{i+1}\right),$$

where sd(·) is the standard deviation. Then, we select the block size $S_{i^{*}}$ with $i^{*}=\arg\min_{i\in \{1,\ldots ,m\}}Y^{i}$.

### 5.2. Simulation Settings

We present the results of a simulation study aimed at evaluating the performance of tests based on $T_{n}$, as defined in (2), in finite samples. To assess the finite-sample properties of the proposed test, we employed the following fundamental generating processes: $W=HA+f\left(a\right)\in R^{n\times p}$, where $A\in R^{p\times p}$ is the loading matrix, $f\left(\cdot \right):R\to R^{n\times p}$ is a constant function, the parameter *a* belongs to the set {0,0.1,0.2,0.3,0.4,0.5,0.6}, representing the distance between the null and alternative hypotheses. Additionally, $H={\left(H_{1},\ldots ,H_{n}\right)}^{T}\in R^{n\times p}$ with $H_{t}=\rho H_{t-1}+\varepsilon_{t}\in R^{p\times 1}$, where $\varepsilon_{t}\overset{iid}{∼}N\left(0_{p},I_{p}\right)$ and ρ∈{0,0.1,0.2}. Construct $f_{i}\left(a\right)={\left(m_{1}^{\left(i\right)},\ldots ,m_{n}^{\left(i\right)}\right)}^{T}\in R^{n\times p}$ such that $m_{t}^{\left(i\right)}={\left(m_{t,1}^{\left(i\right)},\ldots ,m_{t,p}^{\left(i\right)}\right)}^{T}$ for i∈{1,2}, where $m_{t,j}^{\left(1\right)}=a^{j}$ and $m_{t,j}^{\left(2\right)}=a\left(1-j/p\right)$ for each t∈[n] and j∈[p]. Then $f_{1}\left(\cdot \right)$ and $f_{2}\left(\cdot \right)$ represent the sparse and dense signal cases, respectively. We consider three different loading matrices for A as follows:

(M1). Let $V={\left(v_{k,l}\right)}_{1\leq k,l\leq p}$ s.t. $v_{k,l}=0.995^{\left|k-l\right|}$, then let $A=V^{1/2}$.
(M2). Let $A={\left(a_{k,l}\right)}_{1\leq k,l\leq p}$ s.t. $a_{k,k}=1$, $a_{k,l}=0.7$ for |k−l|=1 and $a_{k,l}=0$ otherwise.
(M3). Let r=⌈p/2.5⌉, $V={\left(v_{k,l}\right)}_{1\leq k,l\leq p}$, where $v_{k,k}=1$, $v_{k,l}=0.9$ for r(q−1)+1≤k≠l≤rq with q=1,…,⌊p/r⌋, and $v_{k,l}=0$ otherwise. Let $A=V^{1/2}$.

We assess the finite sample performance of our proposed test (denoted by Yang) in comparison with tests introduced by [5] (denoted by Dempster), [4] (denoted by BS), [6] (denoted by SD), and [8] (denoted by CLX). All tests in our simulations are conducted at the 5% significance level with 1000 Monte Carlo replications, and the number of bootstrap replications is set to 1000. We consider dimensions p∈{50,200,400,800} and sample size pairs $\left(n_{1},n_{2}\right)\in \left\{\left(200,220\right),\left(400,420\right)\right\}$.

### 5.3. Simulation Results

For the testing of the null hypothesis, consider independent generations of $\left\{X_{t}\right\}$ and $\left\{Y_{t}\right\}$, following the same process as W, with identical values for ρ and f(a)=0. The choice of f(a)=0 here is made for the sake of simplicity. We exclusively present the simulation results for (M1) in the main body of the paper. The results obtained for (M2) and (M3) are analogous to those of (M1) and are detailed in the Appendix E.

Table 1 presents the performance of various methods in controlling Type I errors based on (M1). As the dimension *p* or sample size $\left(n_{1},n_{2}\right)$ increases, the results of all methods exhibit small changes, except BS’s. When ρ equals 0, indicating samples are generated from independent Gaussian distributions, both Yang’s method and BS’s method effectively control Type I errors at around 5%, while the control achieved by the other three methods is less optimal. It is noteworthy that, with an increase in ρ, the data generated by the AR(1) model significantly influence the other methods. In contrast, Yang’s method demonstrates superior and more stable results with increasing ρ. These comparative effects are also observable in the results based on (M2) and (M3) in the Appendix E. For this reason, we exclusively compare the empirical power results by different methods with ρ=0.

**Figure 1:**
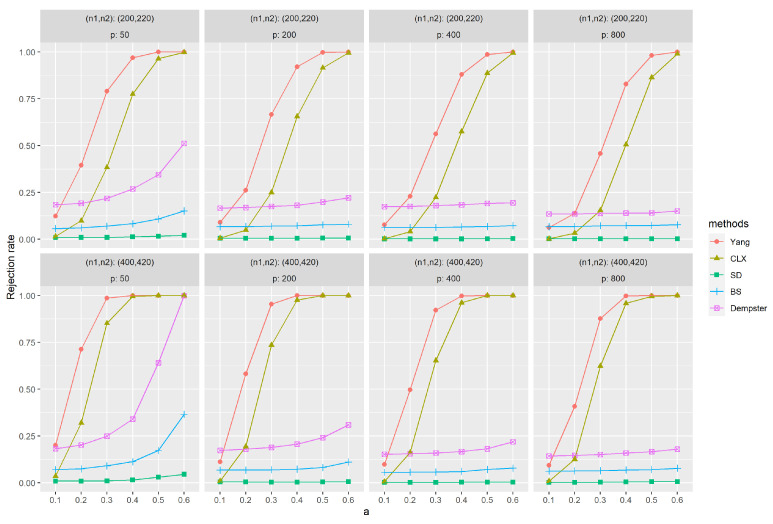
The empirical powers with sparse signals were evaluated by independently generated sequences ${\left\{X_{t}\right\}}_{t=1}^{n_{1}}$ based on (M1), f(·)=0 and ρ=0, and ${\left\{Y_{t}\right\}}_{t=1}^{n_{2}}$ based on (M1), $f\left(\cdot \right)=f_{1}\left(\cdot \right)$ and ρ=0. The parameter *a* represents the distance between the null and alternative hypotheses. The simulations were replicated 1000 times.

**Figure 2:**
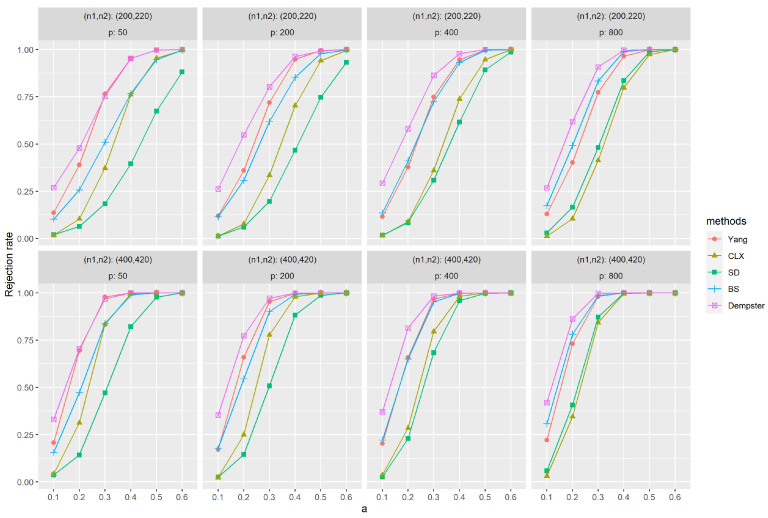
The empirical powers with dense signals were evaluated by independently generated sequences ${\left\{X_{t}\right\}}_{t=1}^{n_{1}}$ based on (M1), f(·)=0 and ρ=0, and ${\left\{Y_{t}\right\}}_{t=1}^{n_{2}}$ based on (M1), $f\left(\cdot \right)=f_{2}\left(\cdot \right)$ and ρ=0. The parameter *a* represents the distance between the null and alternative hypotheses. The simulations were replicated 1000 times.

Figure 1 and Figure 2 depict the empirical power results of various methods for sparse and dense signals based on (M1). Similarly, as the dimension *p* increases, the results of all methods show little variation, except Dempster’s. However, with an increase in sample size $\left(n_{1},n_{2}\right)$, most methods exhibit improvement in their results. In Figure 1, it is evident that Yang’s method outperforms others significantly when the signal is sparse. Methods like SD, BS, and Dempster rely on the $\ell_{2}$-norm of the data, aggregating signals across all dimensions for testing. This makes them less effective when the signal is sparse, i.e., anomalies appear in only a few dimensions. CLX’s approach, akin to Yang’s, tests whether the largest signal is abnormal. Consequently, CLX performs better than the other three methods in scenarios with sparse signals but still falls short of Yang’s method. On the contrary, when the signal is dense, Figure 2 shows that all methods yield favorable results, with Dempster’s method proving to be the most effective. Yang’s method performs at a relatively high level among these methods. In contrast, the CLX’s method, which performs well in sparse signals, exhibits a relatively lower level of performance in dense signals. In conclusion, the proposed method exhibits the most stable performance across all methods and performs exceptionally well on sparse data.

## 6. Real Data Analysis

In this section, we apply the proposed method to a dataset comprised of stock data obtained from Bloomberg’s public database. This dataset includes daily opening prices from 1 January 2018 to 31 December 2021 for 30 companies in the Consumer Discretionary Sector (CDS) and 31 companies in the Information Technology Sector (ITS), all listed in the S&P 500. The sample sizes for the years 2018, 2019, 2020, and 2021 are 251, 250, 253, and 252, respectively. The findings are presented in Table 2. Regarding the data for the Consumer Discretionary (CD) and Information Technology (IT) sectors, all *p*-values from the tests between two consecutive years are 0. This suggests a significant variation in the average annual opening prices across different years for both CDs and ITs.

**Figure 3:**
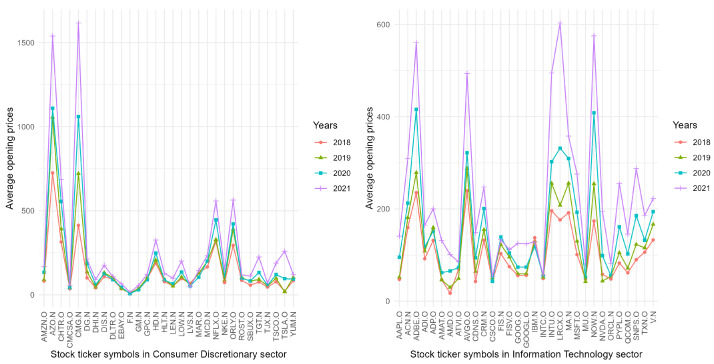
The average annual opening prices of 30 Consumer Discretionary corporations and 31 Information Technology corporations in 2018, 2019, 2020, and 2021.

For data visualization, Figure 3 displays the average annual opening prices of 30 companies in the CDS (left subgragh) and 31 companies in the ITS (right subgragh) in 2018, 2019, 2020, and 2021. The two subgraghs both exhibit a pattern of annual growth in the opening prices of nearly every stock. These results are well in line with the conclusion of Table 2.

## 7. Discussion

In this paper, we propose a two-sample test for high-dimensional time series based on blockwise bootstrap. Our $\ell_{\infty }$-type test statistic is designed to detect the largest abnormal signal among dimensions. Unlike some frameworks, we do not necessarily require independence within each observation or between the two sets of observations. Instead, we rely on the weak dependence property of the pair sequence $\left\{\left(X_{t},Y_{t}\right)\right\}$ to ensure the asymptotic properties of our proposed method. We derive two Gaussian approximation results for two cases in which the dimension *p* diverges, one at a polynomial rate relative to the sample size *n* and the other at an exponential rate relative to the sample size *n*. In the bootstrap procedure, the block size serves as the tuning parameter, and we employ the minimum volatility method, as proposed by [27], for block size selection.

Our test statistic is designed to pinpoint the maximum value among dimensions, facilitating the detection of significant differences in certain dimensions. In cases where differences in each dimension are minimal, it is more appropriate to consider the $\ell_{2}$-type test statistic rather than the $\ell_{\infty }$-type one. Consequently, in the absence of prior information, the utilization of test statistics that combine both types proves advantageous. However, deriving theoretical results from such a combined approach is a significant challenge. As discussed in Section 4, our two-sample testing procedure can be applied to change point detection in high-dimensional time series. The choices of *w*, the size of each subsample mean, and the significance level α play crucial roles in this change point detection procedure. We leave these considerations for future research.

## Figures and Tables

**Table 1:** The Type I error rates, expressed as percentages, were calculated by independently generated sequences ${\left\{X_{t}\right\}}_{t=1}^{n_{1}}$ and ${\left\{Y_{t}\right\}}_{t=1}^{n_{2}}$ based on (M1). The simulations were replicated 1000 times.

($n_{1},n_{2}$)	ρ	p	Yang	Dempster	BS	SD	CLX
(200,220)	0	50	5	18.5	5.8	0.9	0.3
		200	5.9	16.5	6.6	0.4	0.4
		400	5.4	17.4	6.2	0.2	0.3
		800	4.2	13.5	6.7	0.3	0.2
	0.1	50	6.5	22.8	9.3	2	1
		200	6.6	22.6	9.6	1.2	0.8
		400	7.4	22.9	10.4	1	0.8
		800	5.8	22.5	12.4	1	1.2
	0.2	50	6.8	30.2	13.8	3.1	2.5
		200	7.7	29.9	14.3	2.2	2.7
		400	9.3	30.5	18.2	2.2	2.4
		800	7.9	33.3	21.3	3	3.2
(400,420)	0	50	5.2	17.6	6.8	1	0.5
		200	5.3	17.2	6.8	0.5	0.1
		400	4.6	15.1	5.7	0.3	0
		800	5.2	14.2	6.3	0.3	0.4
	0.1	50	5.6	22.4	9.6	1.4	1
		200	6.3	22.5	9.6	1.3	0.8
		400	6.1	21.4	9.7	0.8	0.8
		800	6.5	23.6	12.1	0.7	1.2
	0.2	50	6.7	26.9	12.8	2.5	1.9
		200	7.6	29.2	14.9	2.3	2.4
		400	7.6	29.4	15.1	1.5	2.9
		800	8.3	36.3	21.9	2.5	3.8

**Table 2:** The *p*-values for testing the equality of average annual opening prices across two consecutive years in the Consumer Discretionary Sector and Information Technology Sector, respectively.

Sector of S&P 500	2018–2019	2019–2020	2020–2021
Consumer Discretionary	0	0	0
Information Technology	0	0	0

**Figure A1:**
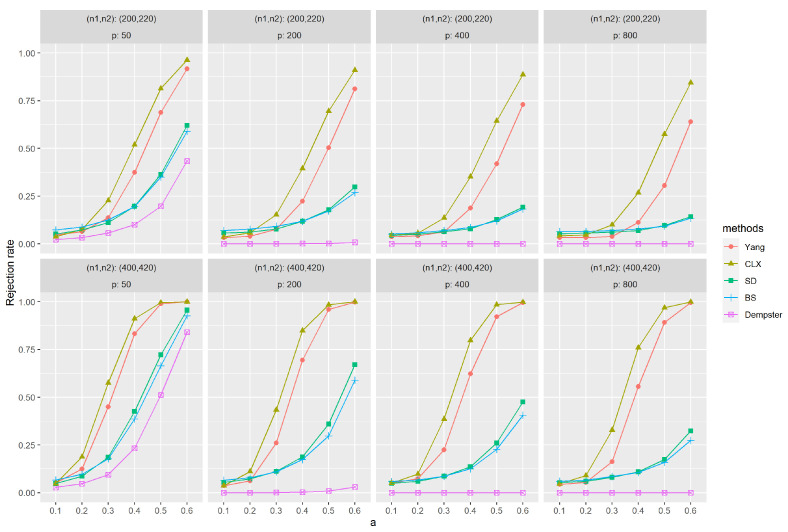
The empirical powers with sparse signals were evaluated by independently generated sequences ${\left\{X_{t}\right\}}_{t=1}^{n_{1}}$ based on (M2), f(·)=0 and ρ=0, and ${\left\{Y_{t}\right\}}_{t=1}^{n_{2}}$ based on (M2), $f\left(\cdot \right)=f_{1}\left(\cdot \right)$ and ρ=0. The parameter *a* represents the distance between the null and alternative hypotheses. The simulations were replicated 1000 times.

**Figure A2:**
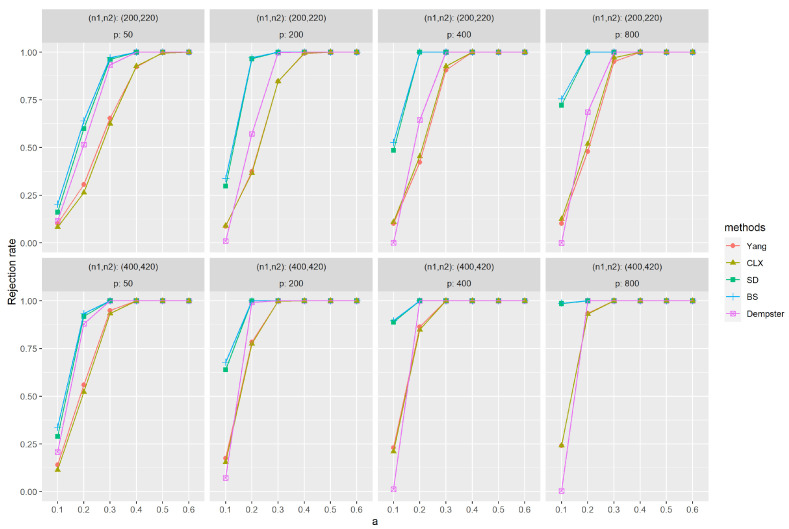
The empirical powers with dense signals were evaluated by independently generated sequences ${\left\{X_{t}\right\}}_{t=1}^{n_{1}}$ based on (M2), f(·)=0 and ρ=0, and ${\left\{Y_{t}\right\}}_{t=1}^{n_{2}}$ based on (M2), $f\left(\cdot \right)=f_{2}\left(\cdot \right)$ and ρ=0. The parameter *a* represents the distance between the null and alternative hypotheses. The simulations were replicated 1000 times.

**Figure A3:**
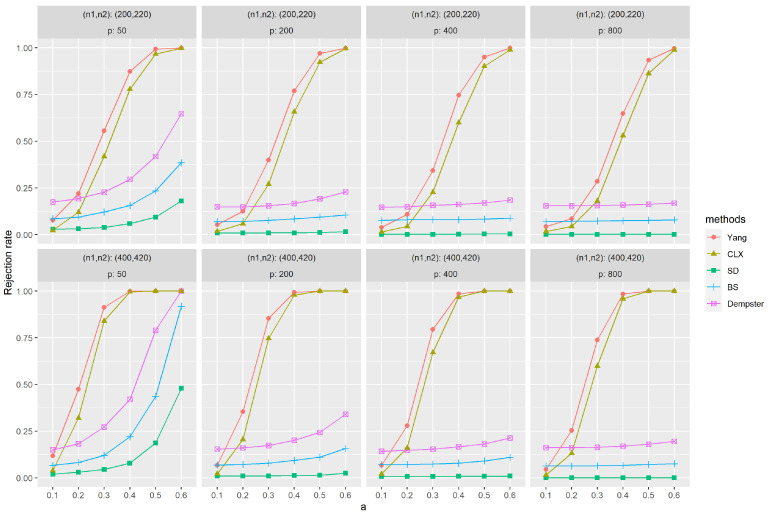
The empirical powers with sparse signals were evaluated by independently generated sequences ${\left\{X_{t}\right\}}_{t=1}^{n_{1}}$ based on (M3), f(·)=0 and ρ=0, and ${\left\{Y_{t}\right\}}_{t=1}^{n_{2}}$ based on (M3), $f\left(\cdot \right)=f_{1}\left(\cdot \right)$ and ρ=0. The parameter *a* represents the distance between the null and alternative hypotheses. The simulations were replicated 1000 times.

**Figure A4:**
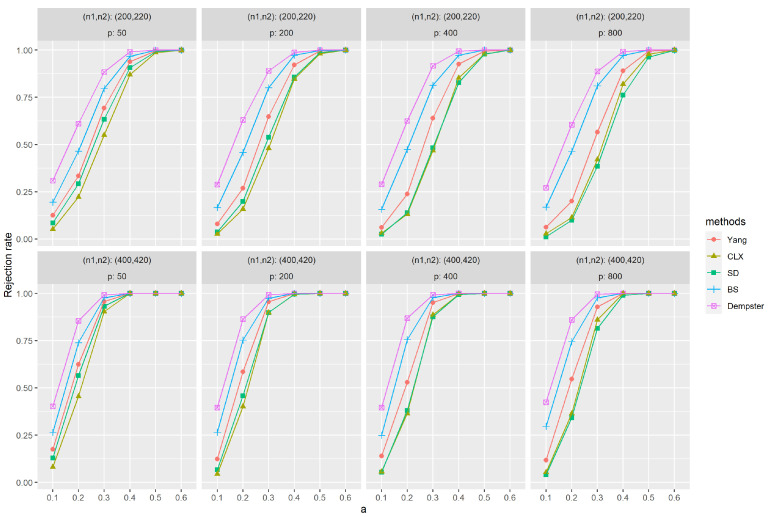
The empirical powers with dense signals were evaluated by independently generated sequences ${\left\{X_{t}\right\}}_{t=1}^{n_{1}}$ based on (M3), f(·)=0 and ρ=0, and ${\left\{Y_{t}\right\}}_{t=1}^{n_{2}}$ based on (M3), $f\left(\cdot \right)=f_{2}\left(\cdot \right)$ and ρ=0. The parameter *a* represents the distance between the null and alternative hypotheses. The simulations were replicated 1000 times.

## Data Availability

The data used to support the findings of this study are included within the article.

## Appendix A. Proof of Theorem 1

### Appendix A.1. Proof of Theorem 1(i)

**Proof.** We first show that, for any τ>(q−1)m/(m−q) with some q∈[2,⌊m⌋],

(A1) $$\begin{array}{c} \max_{j\in [p]}E\left(|\sum_{t=1}^{n}\xi_{t,j}{|}^{q}\right)≲n^{q/2}. \end{array}$$

If q=2, due to $\sum_{\kappa =1}^{\infty }\alpha^{\frac{m-2}{m}}\left(\kappa \right)≲\sum_{\kappa =1}^{\infty }\kappa^{-\frac{(m-2)\tau }{m}}<\infty$, Equation (1.12b) (Davydov’s inequality) of [30] yields

(A2) $$\begin{array}{cc} E\left\{{\left(\sum_{t=1}^{n}\xi_{t,j}\right)}^{2}\right\}= & \;\sum_{t=1}^{n}E(|\xi_{t,j}{|}^{2})+\sum_{t_{1}\neq t_{2}}\mathrm{cov}\left(\xi_{t_{1},j},\xi_{t_{2},j}\right)\\ ≲ & \;n+\sum_{t_{1}\neq t_{2}}\left\{E(\right|\xi_{t_{1},j}{|}^{m}{)\}}^{\frac{1}{m}}\left\{E(\right|\xi_{t_{2},j}{|}^{m}{)\}}^{\frac{1}{m}}\alpha^{\frac{m-2}{m}}\left(\right|t_{1}-t_{2}\left|\right)\\ ≲ & \;n+n\sum_{\kappa =1}^{n}\alpha^{\frac{m-2}{m}}\left(\kappa \right)≲n \end{array}$$

for any j∈[p]. For q>2 and j∈[p], Theorem 6.3 of [30] yields

$$\begin{array}{c} E\left(|\sum_{t=1}^{n}\xi_{t,j}{|}^{q}\right)\leq a_{q}s_{n,j}^{q}+nb_{q}\int_{0}^{1}{\left[\alpha^{-1}\left(u\right)\wedge n\right]}^{q-1}{\left\{\underset{t\in [n]}{\sup}Q_{t,j}\left(u\right)\right\}}^{q}du, \end{array}$$

where $a_{q},b_{q}>0$ are two constants depending only on *q*, $s_{n,j}^{2}=\sum_{t_{1},t_{2}=1}^{n}\left|\mathrm{Cov}\left(\xi_{t_{1},j},\xi_{t_{2},j}\right)\right|$, $\alpha^{-1}\left(u\right)=\sum_{\kappa \geq 0}1\left(u\leq \alpha \left(\kappa \right)\right)$ and $Q_{t,j}\left(u\right)=\inf\{x:P(|\xi_{t,j}\left|>x)\leq u\right\}$. By (A2), it holds that $s_{n,j}^{q}={\left(s_{n,j}^{2}\right)}^{q/2}≲n^{q/2}$. Due to $\max_{t\in [n]}\max_{j\in [p]}E(|\xi_{t,j}{|}^{m})\leq C$, we have $\max_{t\in [n]}\max_{j\in [p]}Q_{t,j}\left(u\right)≲u^{-\frac{1}{m}}$. By the denifition of $\alpha^{-1}\left(\cdot \right)$, we know that $\alpha^{-1}\left(u\right)≲u^{-\frac{1}{\tau }}$. Thus

$$\int_{0}^{1}{\left[\alpha^{-1}\left(u\right)\wedge n\right]}^{q-1}{\left\{\underset{t\in [n]}{\sup}Q_{t,j}\left(u\right)\right\}}^{q}du≲\int_{0}^{1}u^{-\frac{q-1}{\tau }-\frac{q}{m}}du\leq C,$$

where the last inequality follows from τ>(q−1)m/(m−q). Hence, we have

$$E\left(|\sum_{t=1}^{n}\xi_{t,j}{|}^{q}\right)≲n^{q/2}$$

for any j∈[p]. By combining above results, we complete the proof of (A1). Now, we begin to prove Theorem 1(i). Define

$$\begin{array}{c} \omega_{n}=\underset{x>0}{\sup}|P\left(\max_{j\in [p]}S_{n,j}\leq x\right)-P\left(\max_{j\in [p]}W_{j}\leq x\right)|. \end{array}$$

Let ${\overset{ˇ}{S}}_{n}={\left({\overset{ˇ}{S}}_{n,1},\ldots ,{\overset{ˇ}{S}}_{n,2p}\right)}^{T}={\left(S_{n,1},-S_{n,1},\ldots ,S_{n,p},-S_{n,p}\right)}^{T}$ and $\overset{ˇ}{W}={\left({\overset{ˇ}{W}}_{1},\ldots ,{\overset{ˇ}{W}}_{2p}\right)}^{T}={\left(W_{1},-W_{1},\ldots ,W_{p},-W_{p}\right)}^{T}$. Then, we have $\max_{j\in [p]}\left|S_{n,j}\right|=\max_{j\in [2p]}{\overset{ˇ}{S}}_{n,j}$ and $\max_{j\in [p]}\left|W_{j}\right|=\max_{j\in [2p]}{\overset{ˇ}{W}}_{j}$. Then, to obtain Theorem 1(i), without loss of generality, it suffices to specify the convergence rate of $\omega_{n}$. For some constant ς∈(0,1), let $B_{n}=\left\lfloor n^{\varsigma }\right\rfloor$ and $K_{n}=\left\lceil n/B_{n}\right\rceil$ be the number of blocks and the size of each block, respectively. For simplicity, we assume $B_{n}≍n^{\varsigma }$ and $K_{n}=n/B_{n}≍n^{1-\varsigma }$. We first decompose the sequence {1,…,n} into $B_{n}$ blocks: $G_{b}=\left\{\left(b-1\right)K_{n}+1,\ldots ,bK_{n}\right\}$ for $b\in [B_{n}]$. Let $g_{n}\gg k_{n}$ be two non-negative integers such that $K_{n}=g_{n}+k_{n}$. We then decompose each $G_{b}\;\left(b\in \left[B_{n}\right]\right)$ to a “large” block $I_{b}$ with length $g_{n}$ and a “small” block $J_{b}$ with length $k_{n}$: $I_{b}=\left\{\left(b-1\right)K_{n}+1,\ldots ,bK_{n}-k_{n}\right\}$ and $J_{b}=\left\{bK_{n}-k_{n}+1,\ldots ,bK_{n}\right\}$. Let $H_{b}={\left(H_{b,1},\ldots ,H_{b,p}\right)}^{T}=K_{n}^{-1/2}\sum_{t\in I_{b}}\xi_{t}$. For each $b\in [B_{n}]$ and some $D_{n}\to \infty$, define $H_{b}^{+}={\left(H_{b,1}^{+},\ldots ,H_{b,p}^{+}\right)}^{T}$ with $H_{b,j}^{+}=H_{b,j}1(|H_{b,j}|\leq D_{n})-E\{H_{b,j}1(|H_{b,j}|\leq D_{n})\}$ and $H_{b}^{-}={\left(H_{b,1}^{-},\ldots ,H_{b,p}^{-}\right)}^{T}$ with $H_{b,j}^{-}=H_{b,j}1(|H_{b,j}|>D_{n})-E\{H_{b,j}1(|H_{b,j}|>D_{n})\}$. For each j∈[p], by Theorem 2 of [17], there exists an independent sequence ${\left\{{\tilde{H}}_{b,j}\right\}}_{b=1}^{B_{n}}$ such that ${\tilde{H}}_{b,j}$ has the same distribution as $H_{b,j}^{+}$ and

$$\begin{array}{c} E(|{\tilde{H}}_{b,j}-H_{b,j}^{+}\left|\right)≲\int_{0}^{\alpha (k_{n})}\inf\{x\in R:P(|H_{b,j}^{+}|>x)\leq u\}du. \end{array}$$

Due to $|H_{b,j}^{+}|\leq 2D_{n}$, we have $\inf\{x\in R:P(|H_{b,j}^{+}|>x)\leq u\}≲D_{n}$ for any u≥0, which implies

(A3) $$\begin{array}{c} E(|{\tilde{H}}_{b,j}-H_{b,j}^{+}\left|\right)≲D_{n}\alpha \left(k_{n}\right). \end{array}$$

Define ${\tilde{S}}_{n}={\left({\tilde{S}}_{n,1},\ldots ,{\tilde{S}}_{n,p}\right)}^{T}=B_{n}^{-1/2}\sum_{b=1}^{B_{n}}{\tilde{H}}_{b}$ with ${\tilde{S}}_{n,j}=B_{n}^{-1/2}\sum_{b=1}^{B_{n}}{\tilde{H}}_{b,j}$ and

(A4) $$\begin{array}{c} {\tilde{\omega }}_{n}=\underset{x>0}{\sup}|P\left(\max_{j\in [p]}{\tilde{S}}_{n,j}\leq x\right)-P\left(\max_{j\in [p]}W_{j}\leq x\right)|. \end{array}$$

For any $\epsilon_{1}>0$, triangle inequality implies

$$\begin{array}{cc} \; & \;P\left(\max_{j\in [p]}S_{n,j}\leq x\right)\leq P\left(\max_{j\in [p]}{\tilde{S}}_{n,j}\leq x+\epsilon_{1}\right)+P\left(|\max_{j\in [p]}S_{n,j}-\max_{j\in [p]}{\tilde{S}}_{n,j}|>\epsilon_{1}\right)\\ \leq & \;P\left(\max_{j\in [p]}W_{j}\leq x+\epsilon_{1}\right)+{\tilde{\omega }}_{n}+P\left(|\max_{j\in [p]}S_{n,j}-\max_{j\in [p]}{\tilde{S}}_{n,j}|>\epsilon_{1}\right)\\ \leq & \;P\left(\max_{j\in [p]}W_{j}\leq x\right)+P\left(x-\epsilon_{1}<\max_{j\in [p]}W_{j}\leq x+\epsilon_{1}\right)+{\tilde{\omega }}_{n}+P\left(|\max_{j\in [p]}S_{n,j}-\max_{j\in [p]}{\tilde{S}}_{n,j}|>\epsilon_{1}\right) \end{array}$$

for any x>0, then $P\left(\max_{j\in [p]}S_{n,j}\leq x\right)-P\left(\max_{j\in [p]}W_{j}\leq x\right)\leq P\left(x-\epsilon_{1}<\max_{j\in [p]}W_{j}\leq x+\epsilon_{1}\right)+{\tilde{\omega }}_{n}+P(|\max_{j\in [p]}S_{n,j}-\max_{j\in [p]}{\tilde{S}}_{n,j}\left|>\epsilon_{1}\right)$. Likewise, $P\left(\max_{j\in [p]}S_{n,j}\leq x\right)-P\left(\max_{j\in [p]}W_{j}\leq x\right)\geq -P\left(x-\epsilon_{1}<\max_{j\in [p]}W_{j}\leq x+\epsilon_{1}\right)-{\tilde{\omega }}_{n}-P(|\max_{j\in [p]}S_{n,j}-\max_{j\in [p]}{\tilde{S}}_{n,j}\left|>\epsilon_{1}\right)$. Due to $\min_{j\in [p]}{\left(\Sigma_{n}\right)}_{j,j}\geq \lambda_{\min}\left(\Sigma_{n}\right)\geq c$, Lemma A.1 of [31] yields

$$\begin{array}{c} \underset{x\in R}{\sup}P\left(x-\epsilon_{1}<\max_{j\in [p]}W_{j}\leq x+\epsilon_{1}\right)≲\epsilon_{1}{\left(\log p\right)}^{1/2} \end{array}$$

for any $\epsilon_{1}>0$. Thus, we can conclude that

(A5) $$\begin{array}{c} \omega_{n}≲{\tilde{\omega }}_{n}+P\left(|\max_{j\in [p]}S_{n,j}-\max_{j\in [p]}{\tilde{S}}_{n,j}|>\epsilon_{1}\right)+\epsilon_{1}{\left(\log p\right)}^{1/2}. \end{array}$$

Define $S_{n}^{+}={\left(S_{n,1}^{+},\ldots ,S_{n,p}^{+}\right)}^{T}=B_{n}^{-1/2}\sum_{b=1}^{B_{n}}H_{b}^{+}$. By triangle inequality,

$$\begin{array}{c} |\max_{j\in [p]}S_{n,j}-\max_{j\in [p]}S_{n,j}^{+}|\leq \max_{j\in [p]}\left|S_{n,j}-S_{n,j}^{+}\right|\leq \max_{j\in [p]}|\frac{1}{n^{1/2}}\sum_{b=1}^{B_{n}}\sum_{t\in J_{b}}\xi_{t,j}|+\max_{j\in [p]}|\frac{1}{B_{n}^{1/2}}\sum_{b=1}^{B_{n}}H_{b,j}^{-}|. \end{array}$$

By (A1), we have $E(|H_{b,j}{|}^{3})\leq C$. Thus $E(|H_{b,j}^{-}{|}^{3})≲E(|H_{b,j}{|}^{3})\leq C$, and

(A6) $$\begin{array}{c} E(|H_{b,j}^{-}{|}^{2})≲E\{|H_{b,j}{|}^{2}1(|H_{b,j}|>D_{n})\}\leq E(|H_{b,j}{|}^{3})D_{n}^{-1}≲D_{n}^{-1}\;. \end{array}$$

Similar to (A2), we have $E(|\sum_{b=1}^{B_{n}}\sum_{t\in J_{b}}\xi_{t,j}\left|\right)≲B_{n}^{1/2}k_{n}^{1/2}$ for any j∈[p], and

(A7) $$\begin{array}{cc} \; & \;E\left\{{\left(\sum_{b=1}^{B_{n}}H_{b,j}^{-}\right)}^{2}\right\}=\sum_{b=1}^{B_{n}}E(|H_{b,j}^{-}{|}^{2})+\sum_{b_{1}\neq b_{2}}\mathrm{cov}\left(H_{b_{1},j}^{-},H_{b_{2},j}^{-}\right)\\ ≲ & \;B_{n}D_{n}^{-1}+\sum_{b_{1}\neq b_{2}}\alpha^{\frac{1}{3}}\left\{k_{n}1(|b_{1}-b_{2}\left|=1)+\right|b_{2}-b_{1}-1|K_{n}1(|b_{1}-b_{2}\left|>1\right)\right\}\\ ≲ & \;B_{n}D_{n}^{-1}+\sum_{|b_{1}-b_{2}|=1}\alpha^{\frac{1}{3}}\left(k_{n}\right)+\sum_{|b_{1}-b_{2}|>1}\alpha^{\frac{1}{3}}\left(\right|b_{1}-b_{2}-1\left|K_{n}\right)≲B_{n}D_{n}^{-1}+B_{n}k_{n}^{-\frac{\tau }{3}}, \end{array}$$

where the last inequality follows from τ>3. Thus, $E(|\sum_{b=1}^{B_{n}}H_{b,j}^{-}\left|\right)≲B_{n}^{1/2}\left(D_{n}^{-1/2}+k_{n}^{-\tau /6}\right)$ and

(A8) $$\begin{array}{c} E\left(|\max_{j\in [p]}S_{n,j}-\max_{j\in [p]}S_{n,j}^{+}|\right)≲\frac{pk_{n}^{1/2}}{K_{n}^{1/2}}+\frac{p}{D_{n}^{1/2}}+\frac{p}{k_{n}^{\tau /6}}. \end{array}$$

Due to ${\tilde{H}}_{b,j}$ having the same distribution as $H_{b,j}^{+}$ and $|H_{b,j}^{+}|\leq 2D_{n}$, by (A3), we have $E(|{\tilde{H}}_{b,j}-H_{b,j}^{+}{|}^{s})≲D_{n}^{s}k_{n}^{-\tau }$ for s∈{2,3}. Thus, following the same arguments as in the proof of (A7), it holds that

$$\begin{array}{cc} \; & E\left[{\left\{\sum_{b=1}^{B_{n}}\left({\tilde{H}}_{b,j}-H_{b,j}^{+}\right)\right\}}^{2}\right]\\ ≲ & \sum_{b=1}^{B_{n}}D_{n}^{2}k_{n}^{-\tau }+D_{n}^{2}k_{n}^{-2\tau /3}\left\{\sum_{|b_{1}-b_{2}|=1}\alpha^{\frac{1}{3}}\left(k_{n}\right)+\sum_{|b_{1}-b_{2}|>1}\alpha^{\frac{1}{3}}\left(\right|b_{1}-b_{2}-1\left|K_{n}\right)\right\}\\ ≲ & B_{n}D_{n}^{2}k_{n}^{-\tau }. \end{array}$$

Thus, $E(|\sum_{b=1}^{B_{n}}\left({\tilde{H}}_{b,j}-H_{b,j}^{+}\right)\left|\right)≲B_{n}^{1/2}D_{n}k_{n}^{-\tau /2}$ and

$$\begin{array}{c} E\left(|\max_{j\in [p]}{\tilde{S}}_{n,j}-\max_{j\in [p]}S_{n,j}^{+}|\right)\leq E\left(\max_{j\in [p]}\left|{\tilde{S}}_{n,j}-S_{n,j}^{+}\right|\right)≲\frac{pD_{n}}{k_{n}^{\tau /2}}. \end{array}$$

Together with (A8), we have

$$E\left(|\max_{j\in [p]}S_{n,j}-\max_{j\in [p]}{\tilde{S}}_{n,j}|\right)≲\frac{pk_{n}^{1/2}}{K_{n}^{1/2}}+\frac{p}{D_{n}^{1/2}}+\frac{p}{k_{n}^{\tau /6}}+\frac{pD_{n}}{k_{n}^{\tau /2}}.$$

Let $\epsilon_{1}=p^{1/2}{\left(\log p\right)}^{-1/4}{\left(k_{n}^{1/2}K_{n}^{-1/2}+D_{n}^{-1/2}+k_{n}^{-\tau /6}+D_{n}k_{n}^{-\tau /2}\right)}^{1/2}$. It holds by (A5) and Markov inequality that

(A9) $$\begin{array}{c} \omega_{n}≲{\tilde{\omega }}_{n}+p^{1/2}{\left(\log p\right)}^{1/4}{\left(\frac{k_{n}^{1/2}}{K_{n}^{1/2}}+\frac{1}{D_{n}^{1/2}}+\frac{1}{k_{n}^{\tau /6}}+\frac{D_{n}}{k_{n}^{\tau /2}}\right)}^{1/2}. \end{array}$$

Define ${\tilde{\Sigma }}_{G}=B_{n}^{-1}\sum_{b=1}^{B_{n}}\mathrm{var}\left({\tilde{H}}_{b}\right)$ and $\Delta =|\Sigma_{n}-{\tilde{\Sigma }}_{G}|$, where $\Sigma_{n}=E\left(S_{n}S_{n}^{T}\right)$. Note that

(A10) $$\begin{array}{cc} \Delta = & \left|\frac{1}{B_{n}}\sum_{b=1}^{B_{n}}\left\{\mathrm{var}\left({\tilde{H}}_{b}\right)-\mathrm{var}\left(H_{b}^{+}\right)\right\}+\frac{1}{B_{n}}\sum_{b=1}^{B_{n}}\left\{\mathrm{var}\left(H_{b}^{+}\right)-\mathrm{var}\left(H_{b}\right)\right\}+\frac{1}{B_{n}}\sum_{b=1}^{B_{n}}\mathrm{var}\left(H_{b}\right)-\Sigma_{n}\right|\\ \leq & \underset{\Delta_{1}}{\underset{︸}{\frac{1}{B_{n}}\sum_{b=1}^{B_{n}}|\mathrm{var}\left({\tilde{H}}_{b}\right)-\mathrm{var}\left(H_{b}^{+}\right)|}}+\underset{\Delta_{2}}{\underset{︸}{\frac{1}{B_{n}}\sum_{b=1}^{B_{n}}|\mathrm{var}\left(H_{b}^{+}\right)-\mathrm{var}\left(H_{b}\right)|}}+\underset{\Delta_{3}}{\underset{︸}{\left|\frac{1}{B_{n}}\sum_{b=1}^{B_{n}}\mathrm{var}\left(H_{b}\right)-\Sigma_{n}\right|}}. \end{array}$$

In this sequel, we specify the convergence rates of $|\Delta_{1}{|}_{\infty }$, $|\Delta_{2}{|}_{\infty }$, and $|\Delta_{3}{|}_{\infty }$, respectively. Note that the (i,j)-th element of $\mathrm{var}\left({\tilde{H}}_{b}\right)-\mathrm{var}\left(H_{b}^{+}\right)$ is $E({\tilde{H}}_{b,i}{\tilde{H}}_{b,j}-H_{b,i}^{+}H_{b,j}^{+})$. Due to ${\tilde{H}}_{b,j}$ having the same distribution as $H_{b,j}^{+}$ and $|H_{b,j}^{+}|≲D_{n}$ for any $b\in [B_{n}]$ and j∈[p], it holds by (A3) that

$$\begin{array}{c} |E\left({\tilde{H}}_{b,i}{\tilde{H}}_{b,j}-H_{b,i}^{+}H_{b,j}^{+}\right)|\leq |E\left\{\left({\tilde{H}}_{b,i}-H_{b,i}^{+}\right){\tilde{H}}_{b,j}\right\}|+|E\left\{\left({\tilde{H}}_{b,j}-H_{b,j}^{+}\right){\tilde{H}}_{b,i}^{+}\right\}|≲D_{n}^{2}k_{n}^{-\tau } \end{array}$$

for any $b\in [B_{n}]$ and i,j∈[p]. Thus, we can conclude that $|\Delta_{1}{|}_{\infty }≲D_{n}^{2}k_{n}^{-\tau }$. The (i,j)-th element of $\mathrm{var}\left(H_{b}^{+}\right)-\mathrm{var}\left(H_{b}\right)$ is $E(H_{b,i}^{+}H_{b,j}^{+}-H_{b,i}H_{b,j})$. Note that $E(|H_{b,j}^{-}\left|\right)≲E\{|H_{b,j}\left|1(\right|H_{b,j}|>D_{n})\}\leq E(|H_{b,j}{|}^{3})D_{n}^{-2}≲D_{n}^{-2}$. Due to $H_{b,j}=H_{b,j}^{+}+H_{b,j}^{-}$, it holds by (A6) that

$$\begin{array}{cc} |E(H_{b,i}^{+}H_{b,j}^{+}-H_{b,i}H_{b,j})|= & \left|E\{H_{b,i}^{+}H_{b,j}^{+}-\left(H_{b,i}^{+}+H_{b,i}^{-}\right)\left(H_{b,j}^{+}+H_{b,j}^{-}\right)\}\right|\\ \leq & |E\left(H_{b,i}^{+}H_{b,j}^{-}\right)|+|E\left(H_{b,j}^{+}H_{b,i}^{-}\right)|+|E\left(H_{b,i}^{-}H_{b,j}^{-}\right)|≲D_{n}^{-1} \end{array}$$

for any $b\in [B_{n}]$ and i,j∈[p]. Thus, we can conclude that $|\Delta_{2}{|}_{\infty }≲D_{n}^{-1}$. The (i,j)-th element of $\Sigma_{n}-{B_{n}}^{-1}\sum_{b=1}^{B_{n}}\mathrm{var}\left(H_{b}\right)$ is $n^{-1}\sum_{t_{1},t_{2}=1}^{n}E\left(\xi_{t_{1},i}\xi_{t_{2},j}\right)-n^{-1}\sum_{b=1}^{B_{n}}\sum_{t_{1},t_{2}\in I_{b}}E\left(\xi_{t_{1},i}\xi_{t_{2},j}\right)$, and

(A11) $$\begin{array}{cc} \; & \;|\frac{1}{n}\sum_{t_{1},t_{2}=1}^{n}E\left(\xi_{t_{1},i}\xi_{t_{2},j}\right)-\frac{1}{n}\sum_{b=1}^{B_{n}}\sum_{t_{1},t_{2}\in I_{b}}E\left(\xi_{t_{1},i}\xi_{t_{2},j}\right)|\\ = & \;\frac{1}{n}|\sum_{b_{1}\neq b_{2}}E\left\{\left(\sum_{t\in G_{b_{1}}}\xi_{t,i}\right)\left(\sum_{t\in G_{b_{2}}}\xi_{t,j}\right)\right\}\\ \; & \;\;\;\;\;\;\;+\sum_{b=1}^{B_{n}}E\left\{\left(\sum_{t\in I_{b}}\xi_{t,i}\right)\left(\sum_{t\in J_{b}}\xi_{t,j}\right)+\left(\sum_{t\in J_{b}}\xi_{t,i}\right)\left(\sum_{t\in G_{b}}\xi_{t,j}\right)\right\}|. \end{array}$$

Similar to the proof of (A2), we have

$$\begin{array}{cc} \; & \;|E\left\{\left(\sum_{t\in J_{b}}\xi_{t,i}\right)\left(\sum_{t\in G_{b}}\xi_{t,j}\right)\right\}|\\ = & \;|\sum_{t\in J_{b}}\mathrm{cov}\left(\xi_{t,i},\xi_{t,j}\right)+\sum_{t_{1}\neq t_{2}:t_{1},t_{2}\in J_{b}}\mathrm{cov}\left(\xi_{t_{1},i},\xi_{t_{2},j}\right)+\sum_{t_{1}\in J_{b}}\sum_{t_{2}\in I_{b}}\mathrm{cov}\left(\xi_{t_{1},i},\xi_{t_{2},j}\right)|\\ ≲ & \;k_{n}+\sum_{t_{1}\neq t_{2}:t_{1},t_{2}\in J_{b}}\left\{E(\right|\xi_{t_{1},i}{|}^{3}{)\}}^{\frac{1}{3}}\left\{E(\right|\xi_{t_{2},j}{|}^{3}{)\}}^{\frac{1}{3}}\alpha^{\frac{1}{3}}\left(\right|t_{1}-t_{2}\left|\right)\\ \; & \;+\sum_{t_{1}\in J_{b}}\sum_{t_{2}\in I_{b}}\left\{E(\right|\xi_{t_{1},i}{|}^{3}{)\}}^{\frac{1}{3}}\left\{E(\right|\xi_{t_{2},j}{|}^{3}{)\}}^{\frac{1}{3}}\alpha^{\frac{1}{3}}\left(\right|t_{1}-t_{2}\left|\right)≲k_{n}. \end{array}$$

Similarly, we can also obtain

$$|E\left\{\left(\sum_{t\in I_{b}}\xi_{t,i}\right)\left(\sum_{t\in J_{b}}\xi_{t,j}\right)\right\}|≲k_{n}.$$

Thus,

$$\begin{array}{cc} \; & \;|\sum_{b=1}^{B_{n}}E\left\{\left(\sum_{t\in I_{b}}\xi_{t,i}\right)\left(\sum_{t\in J_{b}}\xi_{t,j}\right)+\left(\sum_{t\in J_{b}}\xi_{t,i}\right)\left(\sum_{t\in G_{b}}\xi_{t,j}\right)\right\}|≲k_{n}B_{n}. \end{array}$$

Analogously to the proof of (A2), if $b_{1}<b_{2}$, due to τ>2m/(m−3),

$$\begin{array}{cc} |E\left\{\left(\sum_{t\in G_{b_{1}}}\xi_{t,i}\right)\left(\sum_{t\in G_{b_{2}}}\xi_{t,j}\right)\right\}|≲ & \;\sum_{t_{1}\in G_{b_{1}}}\sum_{t_{2}\in G_{b_{2}}}\left\{E(\right|\xi_{t_{1},i}{|}^{m}{)\}}^{\frac{1}{m}}\left\{E(\right|\xi_{t_{2},i}{|}^{m}{)\}}^{\frac{1}{m}}\alpha^{\frac{m-2}{m}}\left(\right|t_{1}-t_{2}\left|\right)\\ ≲ & \;\sum_{\delta =1}^{K_{n}}\delta \alpha^{\frac{m-2}{m}}\left\{\left(b_{2}-b_{1}-1\right)K_{n}+\delta \right\}\\ ≲ & \;1\left(b_{2}-b_{1}=1\right)+K_{n}^{2}\alpha^{\frac{m-2}{m}}\left\{\left(b_{2}-b_{1}-1\right)K_{n}\right\}1\left(b_{2}-b_{1}>1\right). \end{array}$$

Then,

$$\begin{array}{cc} \; & \;\sum_{b_{1}<b_{2}}|E\left\{\left(\sum_{t\in G_{b_{1}}}\xi_{t,i}\right)\left(\sum_{t\in G_{b_{2}}}\xi_{t,j}\right)\right\}|≲B_{n}+B_{n}K_{n}^{\frac{2m-(m-2)\tau }{m}}\sum_{\delta =1}^{B_{n}}\delta^{\frac{-(m-2)\tau }{m}}≲B_{n}. \end{array}$$

The same result still holds for $b_{1}>b_{2}$. Thus, we can conclude that

$$\begin{array}{cc} \; & \;\sum_{b_{1}\neq b_{2}}|E\left\{\left(\sum_{t\in G_{b_{1}}}\xi_{t,i}\right)\left(\sum_{t\in G_{b_{2}}}\xi_{t,j}\right)\right\}|≲B_{n}. \end{array}$$

Then, by (A11), it holds that

$$\begin{array}{c} |\frac{1}{n}\sum_{t_{1},t_{2}=1}^{n}E\left(\xi_{t_{1},i}\xi_{t_{2},j}\right)-\frac{1}{n}\sum_{b=1}^{B_{n}}\sum_{t_{1},t_{2}\in I_{b}}E\left(\xi_{t_{1},i}\xi_{t_{2},j}\right)|≲\frac{k_{n}}{K_{n}} \end{array}$$

for any i,j∈[p]. Thus, $|\Delta_{3}{|}_{\infty }≲k_{n}K_{n}^{-1}$. By (A10), we can conclude that

$$\begin{array}{c} {\left|\Delta \right|}_{\infty }\leq |\Delta_{1}{|}_{\infty }+|\Delta_{2}{|}_{\infty }+{\left|\Delta_{3}\right|}_{\infty }≲\frac{D_{n}^{2}}{k_{n}^{\tau }}+\frac{1}{D_{n}}+\frac{k_{n}}{K_{n}}. \end{array}$$

Let ${\left\{{\tilde{H}}_{b}^{G}\right\}}_{b=1}^{B_{n}}$ be a sequence of an independent Gaussian vector such that ${\tilde{H}}_{b}^{G}={\left({\tilde{H}}_{b,1}^{G},\ldots ,{\tilde{H}}_{b,p}^{G}\right)}^{T}$$∼N\{0_{p},\mathrm{var}\left({\tilde{H}}_{b}\right)\}$ for each $b\in [B_{n}]$, where ${\tilde{H}}_{b}={\left({\tilde{H}}_{b,1},\ldots ,{\tilde{H}}_{b,p}\right)}^{T}$. By Theorem 1.1 of [15], Cauchy–Schwarz inequality and Jensen’s inequality,

$$\begin{array}{cc} \; & \underset{x>0}{\sup}|P\left(\max_{j\in [p]}\frac{1}{B_{n}^{1/2}}\sum_{b=1}^{B_{n}}{\tilde{H}}_{b,j}\leq x\right)-P\left(\max_{j\in [p]}\frac{1}{B_{n}^{1/2}}\sum_{b=1}^{B_{n}}{\tilde{H}}_{b,j}^{G}\leq x\right)|\\ \; & \;\;\;\;\;\;\;\;\;\;\;≲p^{1/4}\cdot \left\{\sum_{b=1}^{B_{n}}E(|{\tilde{\Sigma }}_{G}^{-1/2}B_{n}^{-1/2}{\tilde{H}}_{b}{|}_{2}^{3})\right\}\\ \; & \;\;\;\;\;\;\;\;\;\;\;≲\frac{p^{1/4}}{B_{n}^{3/2}}{∥{\tilde{\Sigma }}_{G}^{-1/2}∥}_{2}^{3}\cdot \left[\sum_{b=1}^{B_{n}}E\left\{{\left(\sum_{j=1}^{p}{\tilde{H}}_{b,j}^{2}\right)}^{3/2}\right\}\right]\\ \; & \;\;\;\;\;\;\;\;\;\;\;≲\frac{p^{7/4}}{B_{n}^{3/2}}{∥{\tilde{\Sigma }}_{G}^{-1/2}∥}_{2}^{3}\cdot \left\{\sum_{b=1}^{B_{n}}\max_{j\in [p]}E(|{\tilde{H}}_{b,j}{|}^{3})\right\}, \end{array}$$

where ${\tilde{\Sigma }}_{G}=B_{n}^{-1}\sum_{b=1}^{B_{n}}\mathrm{var}\left({\tilde{H}}_{b}\right)$. Note that

$$|\lambda_{\min}\left({\tilde{\Sigma }}_{G}\right)-\lambda_{\min}\left(\Sigma_{n}\right){|\leq ∥\Delta ∥}_{2}\leq p{\left|\Delta \right|}_{\infty }.$$

Due to $\lambda_{\min}\left(\Sigma_{n}\right)\geq c$, we have $\lambda_{\min}\left({\tilde{\Sigma }}_{G}\right)\geq c$ as long as ${p|\Delta |}_{\infty }=o\left(1\right)$. Thus, if ${p|\Delta |}_{\infty }=o\left(1\right)$, we have $∥{\tilde{\Sigma }}_{G}^{-1/2}{∥}_{2}\leq C$. Since $H_{b,j}=K_{n}^{-1/2}\sum_{t\in I_{b}}\xi_{t,j}$, (A1) yields $E(|{\tilde{H}}_{b,j}{|}^{3})=E(|H_{b,j}^{+}{|}^{3})≲E(|H_{b,j}{|}^{3})\leq C$ for any $b\in [B_{n}]$ and j∈[p], which implies

(A12) $$\begin{array}{c} \underset{x>0}{\sup}|P\left(\max_{j\in [p]}\frac{1}{B_{n}^{1/2}}\sum_{b=1}^{B_{n}}{\tilde{H}}_{b,j}\leq x\right)-P\left(\max_{j\in [p]}\frac{1}{B_{n}^{1/2}}\sum_{b=1}^{B_{n}}{\tilde{H}}_{b,j}^{G}\leq x\right)|≲\frac{p^{7/4}}{B_{n}^{1/2}} \end{array}$$

provided that ${p|\Delta |}_{\infty }=o\left(1\right)$. By Proposition 2.1 of [16], we have

(A13) $$\begin{array}{c} \underset{x>0}{\sup}|P\left(\max_{j\in [p]}\frac{1}{B_{n}^{1/2}}\sum_{b=1}^{B_{n}}{\tilde{H}}_{b,j}^{G}\leq x\right)-P\left(\max_{j\in [p]}W_{j}\leq x\right)|≲{\left|\Delta \right|}_{\infty }^{1/2}\log p. \end{array}$$

Then, by (A4), (A12), and (A13), we have

$$\begin{array}{c} {\tilde{\omega }}_{n}≲\frac{p^{7/4}}{B_{n}^{1/2}}+{\left|\Delta \right|}_{\infty }^{1/2}\log p \end{array}$$

provided that ${p|\Delta |}_{\infty }=o\left(1\right)$. Together with (A9),

$$\begin{array}{cc} \omega_{n}≲ & \frac{p^{7/4}}{B_{n}^{1/2}}+{\left|\Delta \right|}_{\infty }^{1/2}\log p+p^{1/2}{\left(\log p\right)}^{1/4}{\left(\frac{k_{n}^{1/2}}{K_{n}^{1/2}}+\frac{1}{D_{n}^{1/2}}+\frac{1}{k_{n}^{\tau /6}}+\frac{D_{n}}{k_{n}^{\tau /2}}\right)}^{1/2} \end{array}$$

provided that ${p|\Delta |}_{\infty }=o\left(1\right)$. Select $D_{n}≍n^{4\tau /(11\tau +12)}$, $k_{n}≍n^{12/(11\tau +12)}$, and ς=7τ/(11τ+12). Then, if $p=o\{n^{2\tau /(11\tau +12)}\}$, we have

$$\begin{array}{c} \omega_{n}≲\frac{p^{7/4}}{n^{7\tau /(22\tau +24)}}+\frac{\log p}{n^{2\tau /(11\tau +12)}}+\frac{p^{1/2}{\left(\log p\right)}^{1/4}}{n^{\tau /(11\tau +12)}}≲\frac{p^{1/2}{\left(\log p\right)}^{1/4}}{n^{\tau /(11\tau +12)}}. \end{array}$$

Hence, we complete the proof of Theorem 1(i). □

### Appendix A.2. Proof of Theorem 1(ii)

**Proof.** Define ${\left\{\left(G_{b},I_{b},J_{b}\right)\right\}}_{b=1}^{B_{n}}$, ${\left\{H_{b}^{+}\right\}}_{b=1}^{B_{n}}$, and ${\left\{H_{b}^{-}\right\}}_{b=1}^{B_{n}}$ in the same manner as in the proof of Theorem 1(i) with $B_{n}≍n^{\varsigma }$, $K_{n}≍n^{1-\varsigma }$, $k_{n}\ll n^{1-\varsigma }$ and $D_{n}\to \infty$, where ς∈(0,1). Let

$$\begin{array}{c} \omega_{n}=\underset{x>0}{\sup}|P\left(\max_{j\in [p]}S_{n,j}\leq x\right)-P\left(\max_{j\in [p]}W_{j}\leq x\right)|. \end{array}$$

Analogously to (A5), due to $\min_{j\in [p]}{\left(\Sigma_{n}\right)}_{j,j}>c$, we have

(A14) $$\begin{array}{c} \omega_{n}≲{\tilde{\omega }}_{n}+P\left(|\max_{j\in [p]}S_{n,j}-\max_{j\in [p]}{\tilde{S}}_{n,j}|>\epsilon_{2}\right)+\epsilon_{2}{\left(\log p\right)}^{1/2} \end{array}$$

for some $\epsilon_{2}>0$, where ${\tilde{S}}_{n,j}=B_{n}^{-1/2}\sum_{b=1}^{B_{n}}{\tilde{H}}_{b,j}$ with $\left\{{\tilde{H}}_{b,j}\right\}$ specified in the same manner as in the proof of Theorem 1(i), and

$$\begin{array}{c} {\tilde{\omega }}_{n}=\underset{x>0}{\sup}|P\left(\max_{j\in [p]}{\tilde{S}}_{n,j}\leq x\right)-P\left(\max_{j\in [p]}W_{j}\leq x\right)|. \end{array}$$

Define $S_{n}^{+}={\left(S_{n,1}^{+},\ldots ,S_{n,p}^{+}\right)}^{T}=B_{n}^{-1/2}\sum_{b=1}^{B_{n}}H_{b}^{+}$. By triangle inequality,

$$\begin{array}{c} |\max_{j\in [p]}S_{n,j}-\max_{j\in [p]}S_{n,j}^{+}|\leq \max_{j\in [p]}\left|S_{n,j}-S_{n,j}^{+}\right|\leq \max_{j\in [p]}|\frac{1}{n^{1/2}}\sum_{b=1}^{B_{n}}\sum_{t\in J_{b}}\xi_{t,j}|+\max_{j\in [p]}|\frac{1}{B_{n}^{1/2}}\sum_{b=1}^{B_{n}}H_{b,j}^{-}|. \end{array}$$

Note that $P(|\xi_{t,j}{M_{n}}^{-1}\left|>x\right)≲\exp\left(-Cx^{\gamma_{1}}\right)$ for any x>0. Let $\tilde{\gamma }={\left(1/\gamma_{1}+1/\gamma_{2}\right)}^{-1}$. By Theorem 1 of [32] and Bonferroni inequality, we have

(A15) $$\begin{array}{c} P\left(\max_{j\in [p]}|\frac{1}{n^{1/2}}\sum_{b=1}^{B_{n}}\sum_{t\in J_{b}}\xi_{t,j}|>x\right)≲pB_{n}k_{n}\exp\left(-\frac{Cn^{\tilde{\gamma }/2}x^{\tilde{\gamma }}}{M_{n}^{\tilde{\gamma }}}\right)+p\exp\left(-\frac{Cnx^{2}}{M_{n}^{2}B_{n}k_{n}}\right) \end{array}$$

for any $x\gg M_{n}n^{-1/2}$. Similarly, by Theorem 1 of [32] again, for any $x\gg M_{n}K_{n}^{-1/2}$,

$$\begin{array}{cc} \; & \;P(|H_{b,j}\left|>x\right)=P\left(|\frac{1}{K_{n}^{1/2}}\sum_{t\in I_{b}}\xi_{t,j}|>x\right)≲K_{n}\exp\left(-\frac{CK_{n}^{\tilde{\gamma }/2}x^{\tilde{\gamma }}}{M_{n}^{\tilde{\gamma }}}\right)+\exp\left(-\frac{Cx^{2}}{M_{n}^{2}}\right). \end{array}$$

Then, if $D_{n}>M_{n}$,

$$\begin{array}{cc} E\{H_{b,j}^{2}1(|H_{b,j}|>D_{n})\}= & \;2\int_{0}^{D_{n}}xP(|H_{b,j}|>D_{n})dx+2\int_{D_{n}}^{\infty }xP(|H_{b,j}\left|>x\right)dx\\ ≲ & \;D_{n}^{2}\left\{K_{n}\exp\left(-\frac{CK_{n}^{\tilde{\gamma }/2}D_{n}^{\tilde{\gamma }}}{M_{n}^{\tilde{\gamma }}}\right)+\exp\left(-\frac{CD_{n}^{2}}{M_{n}^{2}}\right)\right\}\\ \; & \;\;\;\;+K_{n}\int_{D_{n}}^{\infty }x\exp\left(-\frac{CK_{n}^{\tilde{\gamma }/2}x^{\tilde{\gamma }}}{M_{n}^{\tilde{\gamma }}}\right)dx+\int_{D_{n}}^{\infty }x\exp\left(-\frac{Cx^{2}}{M_{n}^{2}}\right)dx\\ ≲ & \;D_{n}^{2}\left\{K_{n}\left(-\frac{CK_{n}^{\tilde{\gamma }/2}D_{n}^{\tilde{\gamma }}}{M_{n}^{\tilde{\gamma }}}\right)+\exp\left(-\frac{CD_{n}^{2}}{M_{n}^{2}}\right)\right\}. \end{array}$$

Thus, for any $b\in [B_{n}]$ and j∈[p],

(A16) $$\begin{array}{cc} \; & \;E(|H_{b,j}^{-}{|}^{2})≲E\{H_{b,j}^{2}1(|H_{b,j}|>D_{n})\}≲D_{n}^{2}\left\{K_{n}\left(-\frac{CK_{n}^{\tilde{\gamma }/2}D_{n}^{\tilde{\gamma }}}{M_{n}^{\tilde{\gamma }}}\right)+\exp\left(-\frac{CD_{n}^{2}}{M_{n}^{2}}\right)\right\}. \end{array}$$

Select $D_{n}=C^{*}M_{n}{\left\{\log\left(pn\right)\right\}}^{1/2}$ for some sufficiently large constant $C^{*}>0$. Thus, for any x≥0,

$$\begin{array}{c} P\left(\max_{j\in [p]}|\frac{1}{B_{n}^{1/2}}\sum_{b=1}^{B_{n}}H_{b,j}^{-}|>x\right)\leq \frac{pB_{n}^{1/2}\max_{j\in [p]}\max_{b\in [B_{n}]}E(|H_{b,j}^{-}\left|\right)}{x}≲\frac{{\left(pn\right)}^{-1}}{x} \end{array}$$

provided that $\log\left(pn\right)=o\left\{K_{n}^{\tilde{\gamma }/\left(2-\tilde{\gamma }\right)}\right\}$. Then, by (A15), we can conclude that for any $x\gg M_{n}n^{-1/2}$,

(A17) $$\begin{array}{c} P\left(|\max_{j\in [p]}S_{n,j}-\max_{j\in [p]}S_{n,j}^{+}|>x\right)≲pB_{n}k_{n}\exp\left(-\frac{Cn^{\tilde{\gamma }/2}x^{\tilde{\gamma }}}{M_{n}^{\tilde{\gamma }}}\right)+p\exp\left(-\frac{Cnx^{2}}{M_{n}^{2}B_{n}k_{n}}\right)+\frac{{\left(pn\right)}^{-1}}{x} \end{array}$$

provided that $\log\left(pn\right)=o\left\{K_{n}^{\tilde{\gamma }/\left(2-\tilde{\gamma }\right)}\right\}$. Similar to (A3), we have

(A18) $$\begin{array}{c} E(|{\tilde{H}}_{b,j}-H_{b,j}^{+}\left|\right)≲D_{n}\alpha \left(k_{n}\right)≲D_{n}\exp\left(-Ck_{n}^{\gamma_{2}}\right)\;. \end{array}$$

Select $k_{n}=C^{**}{\left\{\log\left(pn\right)\right\}}^{1/\gamma_{2}}$ for some sufficiently large constant $C^{**}>0$. By (A18) and triangle inequality,

$$\begin{array}{c} P\left(|\max_{j\in [p]}{\tilde{S}}_{n,j}-\max_{j\in [p]}S_{n,j}^{+}|>x\right)\leq \frac{pB_{n}^{1/2}\max_{b\in [B_{n}]}\max_{j\in [p]}E(|{\tilde{H}}_{b,j}-H_{b,j}^{+}\left|\right)}{x}≲\frac{{\left(pn\right)}^{-1}}{x} \end{array}$$

for any x≥0. Thus, by (A17), for any $x\gg M_{n}n^{-1/2}$,

(A19) $$\begin{array}{c} P\left(|\max_{j\in [p]}S_{n,j}-\max_{j\in [p]}{\tilde{S}}_{n,j}|>x\right)≲pB_{n}k_{n}\exp\left(-\frac{Cn^{\tilde{\gamma }/2}x^{\tilde{\gamma }}}{M_{n}^{\tilde{\gamma }}}\right)+p\exp\left(-\frac{Cnx^{2}}{M_{n}^{2}B_{n}k_{n}}\right)+\frac{{\left(pn\right)}^{-1}}{x} \end{array}$$

provided that $\log\left(pn\right)=o\left\{K_{n}^{\tilde{\gamma }/\left(2-\tilde{\gamma }\right)}\right\}$. Let $\epsilon_{2}=C^{***}M_{n}k_{n}^{1/2}K_{n}^{-1/2}{\left\{\log\left(pn\right)\right\}}^{1/2}$ for some sufficient large constant $C^{***}>0$. It holds by (A14) that

(A20) $$\begin{array}{c} \omega_{n}≲{\tilde{\omega }}_{n}+\frac{M_{n}{\left\{\log\left(pn\right)\right\}}^{\left(2\gamma_{2}+1\right)/2\gamma_{2}}}{K_{n}^{1/2}} \end{array}$$

provided that $\log\left(pn\right)=o\left\{k_{n}^{\tilde{\gamma }/\left(2-\tilde{\gamma }\right)}B_{n}^{\tilde{\gamma }/\left(2-\tilde{\gamma }\right)}\wedge K_{n}^{\tilde{\gamma }/\left(2-\tilde{\gamma }\right)}\right\}$. Define ${\tilde{\Sigma }}_{G}=B_{n}^{-1}\sum_{b=1}^{B_{n}}\mathrm{var}\left({\tilde{H}}_{b}\right)$ and $\Delta =|\Sigma_{n}-{\tilde{\Sigma }}_{G}|$, where $\Sigma_{n}=E\left(S_{n}S_{n}^{T}\right)$. Note that

(A21) $$\begin{array}{cc} \Delta = & \left|\frac{1}{B_{n}}\sum_{b=1}^{B_{n}}\left\{\mathrm{var}\left({\tilde{H}}_{b}\right)-\mathrm{var}\left(H_{b}^{+}\right)\right\}+\frac{1}{B_{n}}\sum_{b=1}^{B_{n}}\left\{\mathrm{var}\left(H_{b}^{+}\right)-\mathrm{var}\left(H_{b}\right)\right\}+\frac{1}{B_{n}}\sum_{b=1}^{B_{n}}\mathrm{var}\left(H_{b}\right)-\Sigma_{n}\right|\\ \leq & \underset{\Delta_{1}}{\underset{︸}{\frac{1}{B_{n}}\sum_{b=1}^{B_{n}}|\mathrm{var}\left({\tilde{H}}_{b}\right)-\mathrm{var}\left(H_{b}^{+}\right)|}}+\underset{\Delta_{2}}{\underset{︸}{\frac{1}{B_{n}}\sum_{b=1}^{B_{n}}|\mathrm{var}\left(H_{b}^{+}\right)-\mathrm{var}\left(H_{b}\right)|}}+\underset{\Delta_{3}}{\underset{︸}{\left|\frac{1}{B_{n}}\sum_{b=1}^{B_{n}}\mathrm{var}\left(H_{b}\right)-\Sigma_{n}\right|}}. \end{array}$$

In this sequel, we will specify the convergence rates of $|\Delta_{1}{|}_{\infty }$, $|\Delta_{2}{|}_{\infty }$ and $|\Delta_{3}{|}_{\infty }$, respectively. Note that the (i,j)-th element of $\mathrm{var}\left({\tilde{H}}_{b}\right)-\mathrm{var}\left(H_{b}^{+}\right)$ is $E({\tilde{H}}_{b,i}{\tilde{H}}_{b,j}-H_{b,i}^{+}H_{b,j}^{+})$. Due to ${\tilde{H}}_{b,j}$ has the same distribution as $H_{b,j}^{+}$ and $|H_{b,j}^{+}|≲D_{n}$ for any $b\in [B_{n}]$ and j∈[p], it holds by (A18) that

$$\begin{array}{c} |E\left({\tilde{H}}_{b,i}{\tilde{H}}_{b,j}-H_{b,i}^{+}H_{b,j}^{+}\right)|\leq |E\left\{\left({\tilde{H}}_{b,i}-H_{b,i}^{+}\right){\tilde{H}}_{b,j}\right\}|+|E\left\{\left({\tilde{H}}_{b,j}-H_{b,j}^{+}\right){\tilde{H}}_{b,i}^{+}\right\}|≲{\left(pn\right)}^{-1} \end{array}$$

for any $b\in [B_{n}]$ and i,j∈[p]. Thus, we can conclude that $|\Delta_{1}{|}_{\infty }≲{\left(pn\right)}^{-1}$. The (i,j)-th element of $\mathrm{var}\left(H_{b}^{+}\right)-\mathrm{var}\left(H_{b}\right)$ is $E(H_{b,i}^{+}H_{b,j}^{+}-H_{b,i}H_{b,j})$. Due to $H_{b,j}=H_{b,j}^{+}+H_{b,j}^{-}$, then it holds by (A16) that

$$\begin{array}{cc} \; & |E\left(H_{b,i}^{+}H_{b,j}^{+}-H_{b,i}H_{b,j}\right)|=|E\left\{H_{b,i}^{+}H_{b,j}^{+}-\left(H_{b,i}^{+}+H_{b,i}^{-}\right)\left(H_{b,j}^{+}+H_{b,j}^{-}\right)\right\}|\\ \leq & |E\left(H_{b,i}^{+}H_{b,j}^{-}\right)|+|E\left(H_{b,j}^{+}H_{b,i}^{-}\right)|+|E\left(H_{b,i}^{-}H_{b,j}^{-}\right)|≲{\left(pn\right)}^{-1} \end{array}$$

for any $b\in [B_{n}]$ and i,j∈[p] provided that $\log\left(pn\right)=o\left\{K_{n}^{\tilde{\gamma }/\left(2-\tilde{\gamma }\right)}\right\}$. Thus, we can conclude that $|\Delta_{2}{|}_{\infty }≲{\left(pn\right)}^{-1}$ provided that $\log\left(pn\right)=o\left\{K_{n}^{\tilde{\gamma }/\left(2-\tilde{\gamma }\right)}\right\}$. The (i,j)-th element of $\Sigma_{n}-{B_{n}}^{-1}\sum_{b=1}^{B_{n}}\mathrm{var}\left(H_{b}\right)$ is $n^{-1}\sum_{t_{1},t_{2}=1}^{n}E\left(\xi_{t_{1},i}\xi_{t_{2},j}\right)-n^{-1}\sum_{b=1}^{B_{n}}\sum_{t_{1},t_{2}\in I_{b}}E\left(\xi_{t_{1},i}\xi_{t_{2},j}\right)$, and

(A22) $$\begin{array}{cc} \; & \;|\frac{1}{n}\sum_{t_{1},t_{2}=1}^{n}E\left(\xi_{t_{1},i}\xi_{t_{2},j}\right)-\frac{1}{n}\sum_{b=1}^{B_{n}}\sum_{t_{1},t_{2}\in I_{b}}E\left(\xi_{t_{1},i}\xi_{t_{2},j}\right)|\\ = & \;\frac{1}{n}|\sum_{b_{1}\neq b_{2}}E\left\{\left(\sum_{t\in G_{b_{1}}}\xi_{t,i}\right)\left(\sum_{t\in G_{b_{2}}}\xi_{t,j}\right)\right\}\\ \; & \;\;\;\;\;\;\;+\sum_{b=1}^{B_{n}}E\left\{\left(\sum_{t\in I_{b}}\xi_{t,i}\right)\left(\sum_{t\in J_{b}}\xi_{t,j}\right)+\left(\sum_{t\in J_{b}}\xi_{t,i}\right)\left(\sum_{t\in G_{b}}\xi_{t,j}\right)\right\}|\;. \end{array}$$

Note that $E(|\xi_{t,j}{|}^{r})≲M_{n}^{r}$ for any constant integer r>0. Equation (1.12b) of [30] yields

(A23) $$\begin{array}{cc} \; & \;|E\left\{\left(\sum_{t\in J_{b}}\xi_{t,i}\right)\left(\sum_{t\in G_{b}}\xi_{t,j}\right)\right\}|\\ = & \;|\sum_{t\in J_{b}}\mathrm{cov}\left(\xi_{t,i},\xi_{t,j}\right)+\sum_{t_{1}\neq t_{2}:t_{1},t_{2}\in J_{b}}\mathrm{cov}\left(\xi_{t_{1},i},\xi_{t_{2},j}\right)+\sum_{t_{1}\in J_{b}}\sum_{t_{2}\in I_{b}}\mathrm{cov}\left(\xi_{t_{1},i},\xi_{t_{2},j}\right)|\\ ≲ & \;M_{n}^{2}k_{n}+\sum_{t_{1}\neq t_{2}:t_{1},t_{2}\in J_{b}}\left\{E(\right|\xi_{t_{1},i}{|}^{3}{)\}}^{\frac{1}{3}}\left\{E(\right|\xi_{t_{2},j}{|}^{3}{)\}}^{\frac{1}{3}}\alpha^{\frac{1}{3}}\left(\right|t_{1}-t_{2}\left|\right)\\ \; & \;+\sum_{t_{1}\in J_{b}}\sum_{t_{2}\in I_{b}}\left\{E(\right|\xi_{t_{1},i}{|}^{3}{)\}}^{\frac{1}{3}}\left\{E(\right|\xi_{t_{2},j}{|}^{3}{)\}}^{\frac{1}{3}}\alpha^{\frac{1}{3}}\left(\right|t_{1}-t_{2}\left|\right)≲M_{n}^{2}k_{n}. \end{array}$$

Similarly, we can also obtain

$$|E\left\{\left(\sum_{t\in I_{b}}\xi_{t,i}\right)\left(\sum_{t\in J_{b}}\xi_{t,j}\right)\right\}|≲M_{n}^{2}k_{n}\;.$$

Thus,

$$\begin{array}{cc} \; & \;|\sum_{b=1}^{B_{n}}E\left\{\left(\sum_{t\in I_{b}}\xi_{t,i}\right)\left(\sum_{t\in J_{b}}\xi_{t,j}\right)+\left(\sum_{t\in J_{b}}\xi_{t,i}\right)\left(\sum_{t\in G_{b}}\xi_{t,j}\right)\right\}|≲M_{n}^{2}k_{n}B_{n}\;. \end{array}$$

By Equation (1.12b) of [30], if $b_{1}<b_{2}$,

$$\begin{array}{cc} \; & \;|E\left\{\left(\sum_{t\in G_{b_{1}}}\xi_{t,i}\right)\left(\sum_{t\in G_{b_{2}}}\xi_{t,j}\right)\right\}|\\ ≲ & \;\sum_{t_{1}\in G_{b_{1}}}\sum_{t_{2}\in G_{b_{2}}}\left\{E(\right|\xi_{t_{1},i}{|}^{3}{)\}}^{\frac{1}{3}}\left\{E(\right|\xi_{t_{2},j}{|}^{3}{)\}}^{\frac{1}{3}}\alpha^{\frac{1}{3}}\left(\right|t_{1}-t_{2}\left|\right)\\ ≲ & \;M_{n}^{2}\sum_{\delta =1}^{K_{n}}\delta \exp\left[-C{\left\{\left(b_{2}-b_{1}-1\right)K_{n}+\delta \right\}}^{\gamma_{2}}\right]\\ ≲ & \;M_{n}^{2}1\left(b_{2}-b_{1}=1\right)+M_{n}^{2}K_{n}^{2}\exp\left\{-C{\left(b_{2}-b_{1}-1\right)}^{\gamma_{2}}{K_{n}}^{\gamma_{2}}\right\}1\left(b_{2}-b_{1}>1\right). \end{array}$$

Thus,

$$\begin{array}{cc} \; & \;\sum_{b_{1}<b_{2}}|E\left\{\left(\sum_{t\in G_{b_{1}}}\xi_{t,i}\right)\left(\sum_{t\in G_{b_{2}}}\xi_{t,j}\right)\right\}|\\ ≲ & \;M_{n}^{2}B_{n}+M_{n}^{2}K_{n}^{2}\sum_{b_{2}-b_{1}=2}^{B_{n}-1}\exp\left\{-C{\left(b_{2}-b_{1}-1\right)}^{\gamma_{2}}{K_{n}}^{\gamma_{2}}\right\}≲M_{n}^{2}B_{n}. \end{array}$$

Same result holds for $b_{1}>b_{2}$. Thus we can conclude that

$$\begin{array}{cc} \; & \;\sum_{b_{1}\neq b_{2}}|E\left\{\left(\sum_{t\in G_{b_{1}}}\xi_{t,i}\right)\left(\sum_{t\in G_{b_{2}}}\xi_{t,j}\right)\right\}|≲M_{n}^{2}B_{n}. \end{array}$$

Note that the above upper bounds do not depend on (i,j). Then by (A22), it holds that $|\Delta_{3}{|}_{\infty }≲M_{n}^{2}k_{n}K_{n}^{-1}$. By (A21), we can conclude that

(A24) $$\begin{array}{c} {\left|\Delta \right|}_{\infty }≲\frac{M_{n}^{2}k_{n}}{K_{n}} \end{array}$$

provided that $\log\left(pn\right)=o\left\{K_{n}^{\tilde{\gamma }/\left(2-\tilde{\gamma }\right)}\right\}$. Let ${\left\{{\tilde{H}}_{b}^{G}\right\}}_{b=1}^{B_{n}}$ be a sequence of independent Gaussian vector such that ${\tilde{H}}_{b}^{G}={\left({\tilde{H}}_{b,1}^{G},\ldots ,{\tilde{H}}_{b,p}^{G}\right)}^{T}∼N\left\{0_{p},\mathrm{var}\left({\tilde{H}}_{b}\right)\right\}$ for any $b\in [B_{n}]$, where ${\tilde{H}}_{b}={\left({\tilde{H}}_{b,1},\ldots ,{\tilde{H}}_{b,p}\right)}^{T}$. Due to $k_{n}≍{\left\{\log\left(pn\right)\right\}}^{1/\gamma_{2}}$, we know that $\min_{j\in [p]}{\left({\tilde{\Sigma }}_{G}\right)}_{j,j}>c$ provided that $M_{n}^{2}{\left\{\log\left(pn\right)\right\}}^{1/\gamma_{2}}=o\left(K_{n}\right)$ and $\log\left(pn\right)=o\left\{K_{n}^{\tilde{\gamma }/\left(2-\tilde{\gamma }\right)}\right\}$. Due to ${\tilde{H}}_{b,j}\leq 2D_{n}≲M_{n}{\left\{\log\left(pn\right)\right\}}^{1/2}$, it holds that $E\left({\tilde{H}}_{b,j}^{4}\right)≲D_{n}^{2}E\left({\tilde{H}}_{b,j}^{2}\right)≲M_{n}^{4}\log\left(pn\right)$ for any $b\in [B_{n}]$ and j∈[p], where the last inequality follows from $E\left({\tilde{H}}_{b,j}^{2}\right)=E(|H_{b,j}^{+}{|}^{2})≲E\left(H_{b,j}^{2}\right)$ and the similar arguments as in the proof of (A23). By Theorem 2.1 of [16], we have

(A25) $$\begin{array}{c} \underset{x>0}{\sup}|P\left(\max_{j\in [p]}{\tilde{S}}_{n,j}\leq x\right)-P\left(\max_{j\in [p]}\frac{1}{B_{n}^{1/2}}\sum_{b=1}^{B_{n}}{\tilde{H}}_{b,j}^{G}\leq x\right)|≲\frac{M_{n}{\left\{\log\left(pn\right)\right\}}^{3/2}}{B_{n}^{1/4}}. \end{array}$$

provided that $M_{n}^{2}{\left\{\log\left(pn\right)\right\}}^{1/\gamma_{2}}=o\left(K_{n}\right)$ and $\log\left(pn\right)=o\left\{K_{n}^{\tilde{\gamma }/\left(2-\tilde{\gamma }\right)}\right\}$. By Proposition 2.1 of [16] and (A24), we have

(A26) $$\begin{array}{cc} \; & \;\underset{x>0}{\sup}|P\left(\max_{j\in [p]}\frac{1}{B_{n}^{1/2}}\sum_{b=1}^{B_{n}}{\tilde{H}}_{b,j}^{G}\leq x\right)-P\left(\max_{j\in [p]}W_{j}\leq x\right)|\\ ≲ & {\;|\Delta |}_{\infty }^{1/2}\log p≲\frac{M_{n}{\left\{\log\left(pn\right)\right\}}^{\left(2\gamma_{2}+1\right)/2\gamma_{2}}}{K_{n}^{1/2}}. \end{array}$$

By (A20), (A25) and (A26), due to $\tilde{\gamma }={\left(1/\gamma_{1}+1/\gamma_{2}\right)}^{-1}$, we have

$$\begin{array}{c} \omega_{n}≲\frac{M_{n}{\left\{\log\left(pn\right)\right\}}^{3/2}}{B_{n}^{1/4}}+\frac{M_{n}{\left\{\log\left(pn\right)\right\}}^{\left(2\gamma_{2}+1\right)/2\gamma_{2}}}{K_{n}^{1/2}} \end{array}$$

provided that $\log\left(pn\right)=o\left\{B_{n}^{\gamma_{1}\gamma_{2}/\left(\gamma_{1}+2\gamma_{2}-\gamma_{1}\gamma_{2}\right)}\wedge K_{n}^{\gamma_{1}\gamma_{2}/\left(2\gamma_{1}+2\gamma_{2}-\gamma_{1}\gamma_{2}\right)}\right\}$ and $M_{n}^{2}{\left\{\log\left(pn\right)\right\}}^{1/\gamma_{2}}=o\left(K_{n}\right)$. Select ς=2/3. Then $B_{n}≍n^{2/3}$, $K_{n}≍n^{1/3}$ and

$$\begin{array}{c} \omega_{n}≲\frac{M_{n}{\left\{\log\left(pn\right)\right\}}^{\max\{\left(2\gamma_{2}+1\right)/2\gamma_{2},3/2\}}}{n^{1/6}} \end{array}$$

provided that $M_{n}^{2}{\left\{\log\left(pn\right)\right\}}^{1/\gamma_{2}}=o\left(n^{1/3}\right)$ and ${\left\{\log\left(pn\right)\right\}}^{3}=o\left\{n^{\gamma_{1}\gamma_{2}/\left(2\gamma_{1}+2\gamma_{2}-\gamma_{1}\gamma_{2}\right)}\right\}$. Thus we complete the proof of Theorem 1(ii). □

## Appendix B. Proof of Proposition 1

**Proof.** Define

$${\overset{˚}{T}}_{n}=|\frac{1}{\sqrt{\tilde{n}}}\sum_{t=1}^{\tilde{n}}{\overset{˚}{Z}}_{t}{|}_{\infty },$$

where ${\overset{˚}{Z}}_{t}=Z_{t}-E\left(Z_{t}\right)$. Under $H_{0}$, we know that $\mu_{X}=\mu_{Y}=:\mu$. Recall $n_{1}≍n_{2}≍n$ and $\Delta_{n}=n_{1}\vee n_{2}-n_{1}\wedge n_{2}$. Without loss of generality, we assume $n_{1}\leq n_{2}$. By triangle inequality, for any j∈[p],

$$\begin{array}{c} |\sum_{t=1}^{\tilde{n}}{\overset{˚}{Z}}_{t,j}-\sum_{t=1}^{\tilde{n}}Z_{t,j}|≲\sum_{t=1}^{n_{1}}|\sqrt{\frac{n_{2}^{2}}{n_{1}\left(n_{1}+n_{2}\right)}}-\sqrt{\frac{n_{1}}{\left(n_{1}+n_{2}\right)}}|+\sum_{t=n_{1}+1}^{n_{2}}|\sqrt{\frac{n_{1}}{\left(n_{1}+n_{2}\right)}}|=O\left(\Delta_{n}\right). \end{array}$$

Thus $|T_{n}-{\overset{˚}{T}}_{n}|=O\left(\Delta_{n}n^{-1/2}\right)$. Write $\delta_{n}=\Delta_{n}n^{-1/2}\pi_{n}$, where $\pi_{n}>0$ diverges at a sufficiently slow rate. Thus, we have

$$\begin{array}{cc} \; & \;P\left(T_{n}\leq x\right)\leq P\left({\overset{˚}{T}}_{n}\leq x+\delta_{n}\right)+P(|T_{n}-{\overset{˚}{T}}_{n}\left|>\delta_{n}\right)\\ \leq & \;P\left(T_{n}^{G}\leq x+\delta_{n}\right)+\underset{x\in R}{\sup}\left|P\left({\overset{˚}{T}}_{n}\leq x\right)-P\left(T_{n}^{G}\leq x\right)\right|+o\left(1\right)\\ \leq & \;P\left(T_{n}^{G}\leq x\right)+\underset{x\in R}{\sup}P\left(x-\delta_{n}\leq T_{n}^{G}\leq x+\delta_{n}\right)+\underset{x\in R}{\sup}\left|P\left({\overset{˚}{T}}_{n}\leq x\right)-P\left(T_{n}^{G}\leq x\right)\right|+o\left(1\right)\;. \end{array}$$

Analogously, we can also obtain that $P\left(T_{n}\leq x\right)\geq P\left(T_{n}^{G}\leq x\right)-\sup_{x\in R}P\left(x-\delta_{n}\leq T_{n}^{G}\leq x+\delta_{n}\right)-\sup_{x\in R}\left|P\left({\overset{˚}{T}}_{n}\leq x\right)-P\left(T_{n}^{G}\leq x\right)\right|-o\left(1\right)$. Thus,

$$\begin{array}{cc} \; & \;\underset{x\in R}{\sup}\left|P\left(T_{n}\leq x\right)-P\left(T_{n}^{G}\leq x\right)\right|\\ \leq & \;\underset{x\in R}{\sup}P\left(x-\delta_{n}\leq T_{n}^{G}\leq x+\delta_{n}\right)+\underset{x\in R}{\sup}\left|P\left({\overset{˚}{T}}_{n}\leq x\right)-P\left(T_{n}^{G}\leq x\right)\right|+o\left(1\right). \end{array}$$

In Case1, by Assumption 1(iii), we have $\min_{j\in [p]}{\left(\Xi_{\tilde{n}}\right)}_{j,j}>c$. Then by Lemma A.1 of [31], due to $\Delta_{n}^{2}\log p=o\left(n\right)$, we have $\sup_{x\in R}P\left(x-\delta_{n}\leq T_{n}^{G}\leq x+\delta_{n}\right)≲\Delta_{n}n^{-1/2}\pi_{n}{\left(\log p\right)}^{1/2}=o\left(1\right)$. By Assumption 1(i), we have $\max_{t\in [\tilde{n}]}\max_{j\in [p]}E(|{\overset{˚}{Z}}_{t,j}{|}^{m})\leq C$. Note that $\Xi_{\tilde{n}}=E\left({\tilde{n}}^{-1/2}\sum_{t=1}^{\tilde{n}}{\overset{˚}{Z}}_{t},{\tilde{n}}^{-1/2}\sum_{t=1}^{\tilde{n}}{\overset{˚}{Z}}_{t}^{T}\right)$. Then by Assumption 1 and Theorem 1(i), due to 3m/(m−4)>max{2m/(m−3),3}, we have $\sup_{x\in R}\left|P\left({\overset{˚}{T}}_{n}\leq x\right)-P\left(T_{n}^{G}\leq x\right)\right|=o\left(1\right)$ provided that $p^{2}\log p=o\left\{n^{4\tau /(11\tau +12)}\right\}$. Thus, if $p^{2}\log p=o\left\{n^{4\tau /(11\tau +12)}\right\}$,

$$\underset{x\in R}{\sup}\left|P\left(T_{n}\leq x\right)-P\left(T_{n}^{G}\leq x\right)\right|=o\left(1\right).$$

Similarly, in Case2, by Assumption 2 and Theorem 1(ii) with $\left(M_{n},\gamma_{1},\gamma_{2}\right)=\left(C,2,1\right)$, we have $\sup_{x\in R}P\left(x-\delta_{n}\leq T_{n}^{G}\leq x+\delta_{n}\right)≲\Delta_{n}n^{-1/2}\pi_{n}{\left(\log p\right)}^{1/2}=o\left(1\right)$ and $\sup_{x\in R}\left|P\left({\overset{˚}{T}}_{n}\leq x\right)-P\left(T_{n}^{G}\leq x\right)\right|=o\left(1\right)$ provided that $\log\left(pn\right)=o\left(n^{1/9}\right)$. Thus, if $\log\left(pn\right)=o\left(n^{1/9}\right)$,

$$\underset{x\in R}{\sup}\left|P\left(T_{n}\leq x\right)-P\left(T_{n}^{G}\leq x\right)\right|=o\left(1\right).$$

We complete the proof of Proposition 1. □

## Appendix C. Proof of Theorem 2

### Appendix C.1. Proof of Theorem 2 under Case3

**Proof.** By Proposition 1 under Case1, it suffices to show

$$\begin{array}{c} \underset{x\in R}{\sup}\left|P\left({\hat{T}}_{n}^{G}\leq x\;|\;E\right)-P\left(T_{n}^{G}\leq x\right)\right|=o_{p}\left(1\right). \end{array}$$

Recall $T_{n}^{G}={\left|G\right|}_{\infty }$ with $G∼N(0,\Xi_{\tilde{n}})$ and ${\hat{T}}_{n}^{G}={\left|{\tilde{n}}^{-1/2}\sum_{t=1}^{\tilde{n}}\left(Z_{t}-\bar{Z}\right)\varrho_{t}^{\prime }\right|}_{\infty }$, where $\Xi_{\tilde{n}}=\mathrm{var}\left({\tilde{n}}^{-1/2}\sum_{t=1}^{\tilde{n}}Z_{t}\right)$. Let

(A27) $$\begin{array}{c} {\hat{\Xi }}_{\tilde{n}}=\frac{1}{\tilde{n}}\sum_{b=1}^{B}\left\{\left(\sum_{t\in I_{b}}\left(Z_{t}-\bar{Z}\right)\right){\left(\sum_{t\in I_{b}}\left(Z_{t}-\bar{Z}\right)\right)}^{T}\right\}. \end{array}$$

By Proposition 2.1 of [16], we have

(A28) $$\begin{array}{c} \underset{x\in R}{\sup}\left|P\left({\hat{T}}_{n}^{G}\leq x\;|\;E\right)-P\left(T_{n}^{G}\leq x\right)\right|≲\Gamma^{1/2}\log p, \end{array}$$

where

$$\Gamma =|\Xi_{\tilde{n}}-{\hat{\Xi }}_{\tilde{n}}{|}_{\infty }=\frac{1}{\tilde{n}}|\sum_{b=1}^{B}\left\{\left(\sum_{t\in I_{b}}\left(Z_{t}-\bar{Z}\right)\right){\left(\sum_{t\in I_{b}}\left(Z_{t}-\bar{Z}\right)\right)}^{T}\right\}-\mathrm{var}\left(\sum_{t=1}^{\tilde{n}}Z_{t}\right){|}_{\infty }.$$

Let ${\overset{˚}{Z}}_{t}={\left({\overset{˚}{Z}}_{t,1},\ldots ,{\overset{˚}{Z}}_{t,p}\right)}^{T}=Z_{t}-E\left(Z_{t}\right)$. Then, for any i,j∈[p], triangle inequality yields

$$\begin{array}{cc} \; & \;|\sum_{b=1}^{B}\left\{\left(\sum_{t\in I_{b}}\left(Z_{t,i}-{\bar{Z}}_{i}\right)\right)\left(\sum_{t\in I_{b}}\left(Z_{t,j}-{\bar{Z}}_{j}\right)\right)\right\}-E\left\{\left(\sum_{t=1}^{\tilde{n}}{\overset{˚}{Z}}_{t,i}\right)\left(\sum_{t=1}^{\tilde{n}}{\overset{˚}{Z}}_{t,j}\right)\right\}|\\ = & \;|\sum_{b=1}^{B}\left\{\left(\sum_{t\in I_{b}}\left({\overset{˚}{Z}}_{t,i}-{\bar{\overset{˚}{Z}}}_{i}\right)\right)\left(\sum_{t\in I_{b}}\left({\overset{˚}{Z}}_{t,j}-{\bar{\overset{˚}{Z}}}_{j}\right)\right)\right\}-E\left\{\left(\sum_{t=1}^{\tilde{n}}{\overset{˚}{Z}}_{t,i}\right)\left(\sum_{t=1}^{\tilde{n}}{\overset{˚}{Z}}_{t,j}\right)\right\}|\\ \leq & \;|\sum_{b=1}^{B}\left\{\left(\sum_{t\in I_{b}}\left({\overset{˚}{Z}}_{t,i}-{\bar{\overset{˚}{Z}}}_{i}\right)\right)\left(\sum_{t\in I_{b}}\left({\overset{˚}{Z}}_{t,j}-{\bar{\overset{˚}{Z}}}_{j}\right)\right)\right\}-\sum_{b=1}^{B}E\left\{\left(\sum_{t\in I_{b}}{\overset{˚}{Z}}_{t,i}\right)\left(\sum_{t\in I_{b}}{\overset{˚}{Z}}_{t,j}\right)\right\}|\\ \; & \;+|\sum_{b=1}^{B}E\left\{\left(\sum_{t\in I_{b}}{\overset{˚}{Z}}_{t,i}\right)\left(\sum_{t\in I_{b}}{\overset{˚}{Z}}_{t,j}\right)\right\}-E\left\{\left(\sum_{t=1}^{\tilde{n}}{\overset{˚}{Z}}_{t,i}\right)\left(\sum_{t=1}^{\tilde{n}}{\overset{˚}{Z}}_{t,j}\right)\right\}|\\ \leq & \;\underset{I_{1,i,j}}{\underset{︸}{\left|\sum_{b=1}^{B}\sum_{t_{1},t_{2}\in I_{b}}\left\{{\overset{˚}{Z}}_{t_{1},i}{\overset{˚}{Z}}_{t_{2},j}-E\left({\overset{˚}{Z}}_{t_{1},i}{\overset{˚}{Z}}_{t_{2},j}\right)\right\}\right|}}+\underset{I_{2,i,j}}{\underset{︸}{\frac{S}{\tilde{n}}|\left(\sum_{t=1}^{\tilde{n}}{\overset{˚}{Z}}_{t,i}\right)\left(\sum_{t=1}^{\tilde{n}}{\overset{˚}{Z}}_{t,j}\right)|}}\\ \; & \;+\underset{I_{3,i,j}}{\underset{︸}{\left|\sum_{b=1}^{B}E\left\{\left(\sum_{t\in I_{b}}{\overset{˚}{Z}}_{t,i}\right)\left(\sum_{t\in I_{b}}{\overset{˚}{Z}}_{t,j}\right)\right\}-E\left\{\left(\sum_{t=1}^{\tilde{n}}{\overset{˚}{Z}}_{t,i}\right)\left(\sum_{t=1}^{\tilde{n}}{\overset{˚}{Z}}_{t,j}\right)\right\}\right|}}. \end{array}$$

In this sequel, we will specify the upper bounds of $I_{1,i,j}$, $I_{2,i,j}$ and $I_{3,i,j}$, respectively. Without loss of generality, we assume $\tilde{n}=BS$ with $B≍n^{\vartheta }$ and $S≍n^{1-\vartheta }$. By Assumption 1(i), it holds that $\max_{t\in [\tilde{n}]}\max_{j\in [p]}E(|{\overset{˚}{Z}}_{t,j}{|}^{m})\leq C$ for some m>4. Then, due to τ>3m/(m−4), (A1) yields

$$E\left({\left[\sum_{t_{1},t_{2}\in I_{b}}\left\{{\overset{˚}{Z}}_{t_{1},i}{\overset{˚}{Z}}_{t_{2},j}-E\left({\overset{˚}{Z}}_{t_{1},i}{\overset{˚}{Z}}_{t_{2},j}\right)\right\}\right]}^{2}\right)\leq E\left\{{\left(\sum_{t\in I_{b}}{\overset{˚}{Z}}_{t,i}\right)}^{2}{\left(\sum_{t\in I_{b}}{\overset{˚}{Z}}_{t,j}\right)}^{2}\right\}≲S^{2}.$$

By triangle inequality,

(A29) $$\begin{array}{cc} \; & E\left({\left[\sum_{b=1}^{B}\sum_{t_{1},t_{2}\in I_{b}}\left\{{\overset{˚}{Z}}_{t_{1},i}{\overset{˚}{Z}}_{t_{2},j}-E\left({\overset{˚}{Z}}_{t_{1},i}{\overset{˚}{Z}}_{t_{2},j}\right)\right\}\right]}^{2}\right)\\ \; & \;\;\;\;\;\;\;\;\;\;\;\;\;\;≲BS^{2}+\sum_{b=1}^{B-1}\sum_{s=1}^{B-b}|\sum_{t_{1},t_{2}\in I_{b}}\sum_{t_{3},t_{4}\in I_{b+s}}\mathrm{cov}\left({\overset{˚}{Z}}_{t_{1},i}{\overset{˚}{Z}}_{t_{2},j},{\overset{˚}{Z}}_{t_{3},i}{\overset{˚}{Z}}_{t_{4},j}\right)|\\ \; & \;\;\;\;\;\;\;\;\;\;\;\;\;\;\leq BS^{2}+\sum_{b=1}^{B-1}\sum_{s=1}^{B-b}|\sum_{t_{1},t_{2}\in I_{b}}\sum_{t_{3},t_{4}\in I_{b+s}}{\mathrm{cum}}_{i,j}\left(t_{2}-t_{1},t_{3}-t_{1},t_{4}-t_{1}\right)|\\ \; & \;\;\;\;\;\;\;\;\;\;\;\;\;\;\;\;\;+\sum_{b=1}^{B-1}\sum_{s=1}^{B-b}|\sum_{t_{1},t_{2}\in I_{b}}\sum_{t_{3},t_{4}\in I_{b+s}}E\left({\overset{˚}{Z}}_{t_{1},i}{\overset{˚}{Z}}_{t_{3},i}\right)E\left({\overset{˚}{Z}}_{t_{2},j}{\overset{˚}{Z}}_{t_{4},j}\right)|\\ \; & \;\;\;\;\;\;\;\;\;\;\;\;\;\;\;\;\;+\sum_{b=1}^{B-1}\sum_{s=1}^{B-b}|\sum_{t_{1},t_{2}\in I_{b}}\sum_{t_{3},t_{4}\in I_{b+s}}E\left({\overset{˚}{Z}}_{t_{1},i}{\overset{˚}{Z}}_{t_{4},j}\right)E\left({\overset{˚}{Z}}_{t_{3},i}{\overset{˚}{Z}}_{t_{2},j}\right)\}|. \end{array}$$

By Assumption 3, $\sum_{t_{1},t_{2}\in I_{b}}\sum_{t_{3},t_{4}\in I_{b+s}}{\mathrm{cum}}_{i,j}\left(t_{2}-t_{1},t_{3}-t_{1},t_{4}-t_{1}\right)≲S$, which implies

(A30) $$\begin{array}{c} \sum_{b=1}^{B-1}\sum_{s=1}^{B-b}|\sum_{t_{1},t_{2}\in I_{b}}\sum_{t_{3},t_{4}\in I_{b+s}}{\mathrm{cum}}_{i,j}\left(t_{2}-t_{1},t_{3}-t_{1},t_{4}-t_{1}\right)|≲B^{2}S. \end{array}$$

For any b∈[B−1] and s∈[B−b], due to τ>3m/(m−4), Equation (1.12b) of [30] yields

$$\begin{array}{cc} \; & \;|\sum_{t_{1}\in I_{b}}\sum_{t_{3}\in I_{b+s}}E\left({\overset{˚}{Z}}_{t_{1},i}{\overset{˚}{Z}}_{t_{3},i}\right)|\leq \sum_{t_{1}\in I_{b}}\sum_{t_{3}\in I_{b+s}}\left|E\left({\overset{˚}{Z}}_{t_{1},i}{\overset{˚}{Z}}_{t_{3},i}\right)\right|\\ ≲ & \;\sum_{t_{1}\in I_{b}}\sum_{t_{3}\in I_{b+s}}\left\{E(\right|Z_{t_{1},i}{|}^{m}{)\}}^{\frac{1}{m}}\left\{E(\right|Z_{t_{3},i}{|}^{m}{)\}}^{\frac{1}{m}}\alpha^{\frac{m-2}{m}}\left(t_{3}-t_{1}\right)≲\sum_{t_{1}\in I_{b}}\sum_{t_{3}\in I_{b+s}}\alpha^{\frac{m-2}{m}}\left(t_{3}-t_{1}\right)\\ ≲ & \;\sum_{h=1}^{S}h\alpha^{\frac{m-2}{m}}\left\{h+\left(s-1\right)S\right\}≲1\left(s=1\right)+S^{\frac{2m-(m-2)\tau }{m}}{\left(s-1\right)}^{-\frac{(m-2)\tau }{m}}1\left(s>1\right). \end{array}$$

Similarly, we also have

$$|\sum_{t_{2}\in I_{b}}\sum_{t_{4}\in I_{b+s}}E\left({\overset{˚}{Z}}_{t_{2},j}{\overset{˚}{Z}}_{t_{4},j}\right)|≲1\left(s=1\right)+S^{\frac{2m-(m-2)\tau }{m}}{\left(s-1\right)}^{-\frac{(m-2)\tau }{m}}1\left(s>1\right).$$

Thus,

(A31) $$\begin{array}{cc} \; & \;\sum_{b=1}^{B-1}\sum_{s=1}^{B-b}|\sum_{t_{1},t_{2}\in I_{b}}\sum_{t_{3},t_{4}\in I_{b+s}}E\left({\overset{˚}{Z}}_{t_{1},i}{\overset{˚}{Z}}_{t_{3},i}\right)E\left({\overset{˚}{Z}}_{t_{2},j}{\overset{˚}{Z}}_{t_{4},j}\right)|\\ ≲ & \;\sum_{b=1}^{B-1}\sum_{s=1}^{B-b}\left\{1\left(s=1\right)+S^{\frac{4m-2(m-2)\tau }{m}}{\left(s-1\right)}^{-\frac{2(m-2)\tau }{m}}1\left(s>1\right)\right\}≲B. \end{array}$$

Analogously, we also have $\sum_{b=1}^{B-1}\sum_{s=1}^{B-b}|\sum_{t_{1},t_{2}\in I_{b}}\sum_{t_{3},t_{4}\in I_{b+s}}E\left({\overset{˚}{Z}}_{t_{1},i}{\overset{˚}{Z}}_{t_{4},j}\right)E\left({\overset{˚}{Z}}_{t_{3},i}{\overset{˚}{Z}}_{t_{2},j}\right)\}|≲B$. Combining this with (A29)–(A31), due to B≥S,

$$E\left({\left[\sum_{b=1}^{B}\sum_{t_{1},t_{2}\in I_{b}}\left\{{\overset{˚}{Z}}_{t_{1},i}{\overset{˚}{Z}}_{t_{2},j}-E\left({\overset{˚}{Z}}_{t_{1},i}{\overset{˚}{Z}}_{t_{2},j}\right)\right\}\right]}^{2}\right)≲B^{2}S.$$

Then it holds that

(A32) $$\begin{array}{c} I_{1,i,j}=O_{p}\left(BS^{1/2}\right). \end{array}$$

Similar to (A1), we have $|\left(\sum_{t=1}^{\tilde{n}}{\overset{˚}{Z}}_{t,i}\right)\left(\sum_{t=1}^{\tilde{n}}{\overset{˚}{Z}}_{t,j}\right)|=O_{p}\left(n\right)$. Thus, we know that

(A33) $$\begin{array}{c} I_{2,i,j}=O_{p}\left(S\right). \end{array}$$

Note that

$$\begin{array}{c} I_{3,i,j}\leq \sum_{b_{1}\neq b_{2}}|E\left\{\left(\sum_{t\in I_{b_{1}}}{\overset{˚}{Z}}_{t,i}\right)\left(\sum_{t\in I_{b_{2}}}{\overset{˚}{Z}}_{t,j}\right)\right\}|. \end{array}$$

For $b_{1}<b_{2}$, due to τ>3m/(m−4), Equation (1.12b) of [30] yields

$$\begin{array}{cc} \; & \;|E\left\{\left(\sum_{t\in I_{b_{1}}}{\overset{˚}{Z}}_{t,i}\right)\left(\sum_{t\in I_{b_{2}}}{\overset{˚}{Z}}_{t,j}\right)\right\}|\\ ≲ & \;\sum_{s=1}^{S}s\{E(|Z_{t,i}{|}^{m}{)\}}^{\frac{1}{m}}\left\{E(\right|Z_{t+s,j}{|}^{m}{)\}}^{\frac{1}{m}}\alpha^{\frac{m-2}{m}}\left\{s+\left(b_{2}-b_{1}-1\right)S\right\}\\ ≲ & \;1\left(b_{2}-b_{1}=1\right)+S^{\frac{2m-(m-2)\tau }{m}}{\left(b_{2}-b_{1}-1\right)}^{-\frac{(m-2)\tau }{m}}1\left(b_{2}-b_{1}>1\right). \end{array}$$

Thus,

$$\begin{array}{cc} \sum_{b_{1}<b_{2}}|E\left\{\left(\sum_{t\in I_{b_{1}}}{\overset{˚}{Z}}_{t,i}\right)\left(\sum_{t\in I_{b_{2}}}{\overset{˚}{Z}}_{t,j}\right)\right\}|≲ & \;B+S^{\frac{2m-(m-2)\tau }{m}}\sum_{b_{2}-b_{1}>1}{\left(b_{2}-b_{1}-1\right)}^{-\frac{(m-2)\tau }{m}}≲B. \end{array}$$

Similarly, we can also obtain $\sum_{b_{1}>b_{2}}\left|E\left\{\left(\sum_{t\in I_{b_{1}}}{\overset{˚}{Z}}_{t,i}\right)\left(\sum_{t\in I_{b_{2}}}{\overset{˚}{Z}}_{t,j}\right)\right\}\right|≲B$, which implies $I_{3,i,j}≲B$. Then by (A32) and (A33), it holds that

$$\frac{1}{\tilde{n}}|\sum_{b=1}^{B}\left(\sum_{t\in I_{b}}\left(Z_{t,i}-{\bar{Z}}_{i}\right)\right)\left(\sum_{t\in I_{b}}\left(Z_{t,j}-{\bar{Z}}_{j}\right)\right)-E\left\{\left(\sum_{t=1}^{\tilde{n}}{\overset{˚}{Z}}_{t,i}\right)\left(\sum_{t=1}^{\tilde{n}}{\overset{˚}{Z}}_{t,j}\right)\right\}|=O_{p}\left(S^{-1/2}\right).$$

Then, by Markov’s inequality,

(A34) $$\begin{array}{c} \Gamma =|\Xi_{\tilde{n}}-{\hat{\Xi }}_{\tilde{n}}{|}_{\infty }=O_{p}\left(p^{2}S^{-1/2}\right). \end{array}$$

By (A28), due to $S≍n^{1-\vartheta }$, it holds that

(A35) $$\begin{array}{c} \underset{x\in R}{\sup}\left|P\left({\hat{T}}_{n}^{G}\leq x\;|\;E\right)-P\left(T_{n}^{G}\leq x\right)\right|=o_{p}\left(1\right) \end{array}$$

provided that $p\log p=o\{n^{(1-\vartheta )/4}\}$. Recall ${\hat{\mathrm{cv}}}_{\alpha }=\inf\left\{x>0:P\left({\hat{T}}_{n}^{G}>x\;|\;E\right)\leq \alpha \right\}$. For any ϵ>0, let ${\mathrm{cv}}_{\alpha }^{\left(\epsilon \right)}$ and ${\mathrm{cv}}_{\alpha }^{\left(-\epsilon \right)}$ be two constants which satisfy $P\{T_{n}^{G}>{\mathrm{cv}}_{\alpha }^{\left(\epsilon \right)}\}=\alpha +\epsilon$ and $P\{T_{n}^{G}>{\mathrm{cv}}_{\alpha }^{\left(-\epsilon \right)}\}=\alpha -\epsilon$, respectively. We claim that for any ϵ>0, it holds that $P\{{\mathrm{cv}}_{\alpha }^{\left(\epsilon \right)}<{\hat{\mathrm{cv}}}_{\alpha }<{\mathrm{cv}}_{\alpha }^{\left(-\epsilon \right)}\}\to 1$ as n→∞. Otherwise, if ${\hat{\mathrm{cv}}}_{\alpha }\leq {\mathrm{cv}}_{\alpha }^{\left(\epsilon \right)}$, by (A35), we have

$$\begin{array}{c} \alpha =P\left({\hat{T}}_{n}^{G}>{\hat{\mathrm{cv}}}_{\alpha }\;|\;E\right)\geq P\left\{{\hat{T}}_{n}^{G}>{\mathrm{cv}}_{\alpha }^{\left(\epsilon \right)}\;|\;E\right\}=P\left\{T_{n}^{G}>{\mathrm{cv}}_{\alpha }^{\left(\epsilon \right)}\right\}+o_{p}\left(1\right)=\alpha +\epsilon +o_{p}\left(1\right), \end{array}$$

which is a contradiction with probability approaching one as n→∞. Analogously, if ${\hat{\mathrm{cv}}}_{\alpha }\geq {\mathrm{cv}}_{\alpha }^{\left(-\epsilon \right)}$, by (A35), we have

$$\begin{array}{c} \alpha =P\left({\hat{T}}_{n}^{G}>{\hat{\mathrm{cv}}}_{\alpha }\;|\;E\right)\leq P\left\{{\hat{T}}_{n}^{G}>{\mathrm{cv}}_{\alpha }^{\left(-\epsilon \right)}\;|\;E\right\}=P\left\{T_{n}^{G}>{\mathrm{cv}}_{\alpha }^{\left(-\epsilon \right)}\right\}+o_{p}\left(1\right)=\alpha -\epsilon +o_{p}\left(1\right), \end{array}$$

which is also a contradiction with probability approaching one as n→∞. For any ϵ>0, define the event $E_{1,\epsilon }=\left\{{\mathrm{cv}}_{\alpha }^{\left(\epsilon \right)}<{\hat{\mathrm{cv}}}_{\alpha }<{\mathrm{cv}}_{\alpha }^{\left(-\epsilon \right)}\right\}$. Then $P(E_{1,\epsilon })\to 1$ as n→∞. On the one hand, by Proposition 1,

$$\begin{array}{cc} P(T_{n}>{\hat{\mathrm{cv}}}_{\alpha })\leq & \;P\left(T_{n}>{\hat{\mathrm{cv}}}_{\alpha }\;|\;E_{1,\epsilon }\right)+P\left(E_{1,\epsilon }^{c}\right)\leq P\left\{T_{n}>{\mathrm{cv}}_{\alpha }^{\left(\epsilon \right)}\right\}+o\left(1\right)\\ = & \;P\left\{T_{n}^{G}>{\mathrm{cv}}_{\alpha }^{\left(\epsilon \right)}\right\}+o\left(1\right)=\alpha +\epsilon +o\left(1\right), \end{array}$$

which implies that ${\bar{\lim}}_{n\to \infty }P\left(T_{n}>{\hat{\mathrm{cv}}}_{\alpha }\right)\leq \alpha +\epsilon$. On the other hand, by Proposition 1,

$$\begin{array}{cc} P(T_{n}>{\hat{\mathrm{cv}}}_{\alpha })\geq & \;P\left(T_{n}>{\hat{\mathrm{cv}}}_{\alpha }\;|\;E_{1,\epsilon }\right)\geq P\left\{T_{n}>{\mathrm{cv}}_{\alpha }^{\left(-\epsilon \right)}\right\}-P\left(E_{1,\epsilon }^{c}\right)\\ \geq & \;P\left\{T_{n}^{G}>{\mathrm{cv}}_{\alpha }^{\left(-\epsilon \right)}\right\}-o\left(1\right)=\alpha -\epsilon -o\left(1\right), \end{array}$$

which implies that ${\underline{\lim}}_{n\to \infty }P\left(T_{n}>{\hat{\mathrm{cv}}}_{\alpha }\right)\geq \alpha -\epsilon$. Since $P(T_{n}>{\hat{\mathrm{cv}}}_{\alpha })$ does not depend on ϵ, by letting $\epsilon \to 0^{+}$, we have $\lim_{n\to \infty }P\left(T_{n}>{\hat{\mathrm{cv}}}_{\alpha }\right)=\alpha$. Thus we complete the proof of Theorem 2 under Case3. □

### Appendix C.2. Proof of Theorem 2 under Case4

**Proof.** By Proposition 1 under Case2 and the arguments in Appendix C.1, it suffices to show

$$\begin{array}{c} \underset{x\in R}{\sup}\left|P\left({\hat{T}}_{n}^{G}\leq x\;|\;E\right)-P\left(T_{n}^{G}\leq x\right)\right|≲\Gamma^{1/2}\log p=o_{p}\left(1\right), \end{array}$$

where

$$\Gamma \leq \max_{i,j\in [p]}{\tilde{n}}^{-1}|I_{1,i,j}|+\max_{i,j\in [p]}{\tilde{n}}^{-1}|I_{2,i,j}|+\max_{i,j\in [p]}{\tilde{n}}^{-1}\left|I_{3,i,j}\right|$$

with $I_{1,i,j}$, $I_{2,i,j}$ and $I_{3,i,j}$ specified in Appendix C.1. In this sequel, we will specify the upper bounds of $\max_{i,j\in [p]}\left|I_{1,i,j}\right|$, $\max_{i,j\in [p]}\left|I_{2,i,j}\right|$ and $\max_{i,j\in [p]}\left|I_{3,i,j}\right|$, respectively. Without loss of generality, we assume $\tilde{n}=BS$ with $B≍{\tilde{n}}^{\vartheta }$ and $S≍{\tilde{n}}^{1-\vartheta }$ for some ϑ∈[1/2,1). Let $W_{b,i,j}=\sum_{t_{1},t_{2}\in I_{b}}{\overset{˚}{Z}}_{t_{1},i}{\overset{˚}{Z}}_{t_{2},j}-E\left(\sum_{t_{1},t_{2}\in I_{b}}{\overset{˚}{Z}}_{t_{1},i}{\overset{˚}{Z}}_{t_{2},j}\right)$. For $R_{n}>C^{*}S$ with some sufficiently large constant $C^{*}>0$, denote $W_{b,i,j}^{+}=W_{b,i,j}1(|W_{b,i,j}|\leq R_{n})-E\{W_{b,i,j}1(|W_{b,i,j}|\leq R_{n})\}$ and $W_{b,i,j}^{-}=W_{b,i,j}1(|W_{b,i,j}|>R_{n})-E\{W_{b,i,j}1(|W_{b,i,j}|>R_{n})\}$. Then for some $C_{n}>0$, it holds by Bonferroni inequality that

$$\begin{array}{cc} P\left(\max_{i,j\in [p]}|\sum_{b=1}^{B}W_{b,i,j}|>\tilde{n}x\right)\leq & \;p^{2}\max_{i,j\in [p]}P\left(|\sum_{b=1}^{B}W_{b,i,j}^{+}|+|\sum_{b=1}^{B}W_{b,i,j}^{-}|>\tilde{n}x\right)\\ \leq & \;p^{2}\max_{i,j\in [p]}\left\{P\left(|\sum_{b=1}^{B}W_{b,i,j}^{+}|>\tilde{n}x-C_{n}\right)+P\left(|\sum_{b=1}^{B}W_{b,i,j}^{-}|>C_{n}\right)\right\} \end{array}$$

for all $x>C_{n}{\tilde{n}}^{-1}$. Note that

$$\begin{array}{c} E\{W_{b,i,j}^{2}1(|W_{b,i,j}|>R_{n})\}=2\int_{0}^{R_{n}}xP(|W_{b,i,j}|>R_{n})dx+2\int_{R_{n}}^{\infty }xP(|W_{b,i,j}\left|>x\right)dx \end{array}$$

By Assumptions 2(i)–(ii) and Cauchy–Schwarz inequality, $E(\sum_{t_{1},t_{2}\in I_{b}}{\overset{˚}{Z}}_{t_{1},i}{\overset{˚}{Z}}_{t_{2},j})≲S$. By Assumptions 2(i)–(ii) again and Theorem 1 of [32], we know that

$$P\left(|\sum_{t\in I_{b}}{\overset{˚}{Z}}_{t,i}|>x\right)≲S\exp\left(-Cx^{2/3}\right)+\exp\left(-CS^{-1}x^{2}\right)$$

for any x→∞. Thus, for any x>CS, we have

$$\begin{array}{cc} \; & \;P(|W_{b,i,j}\left|>x\right)\leq P\left(|\sum_{t\in I_{b}}{\overset{˚}{Z}}_{t,i}||\sum_{t\in I_{b}}{\overset{˚}{Z}}_{t,j}|>Cx\right)\\ \leq & \;P\left(|\sum_{t\in I_{b}}{\overset{˚}{Z}}_{t,i}|>Cx^{1/2}\right)+P\left(|\sum_{t\in I_{b}}{\overset{˚}{Z}}_{t,j}|>Cx^{1/2}\right)≲S\exp\left(-Cx^{1/3}\right)+\exp\left(-CS^{-1}x\right). \end{array}$$

Due to $R_{n}>CS$, we can show that

$$\begin{array}{cc} \; & \;E(|W_{b,i,j}^{-}{|}^{2})≲E\{W_{b,i,j}^{2}1(|W_{b,i,j}|>R_{n})\}≲R_{n}^{2}S\exp\left(-CR_{n}^{1/3}\right)+R_{n}^{2}\exp\left(-CS^{-1}R_{n}\right). \end{array}$$

Selecting $R_{n}=C^{**}S\log\left(pn\right)$ for some sufficiently large constant $C^{**}>0$, and $C_{n}≍B^{1/2}$, it holds by Markov’s inequality that

$$\begin{array}{c} p^{2}\max_{i,j\in [p]}P\left(|\sum_{b=1}^{B}W_{b,i,j}^{-}|>C_{n}\right)≲p^{2}B^{1/2}\max_{i,j\in [p]}\max_{b\in [B]}E(|W_{b,i,j}^{-}\left|\right)=o\left(1\right) \end{array}$$

provided that $\log\left(pn\right)=o\left(S^{1/2}\right)$. Due to $|W_{b,i,j}^{+}|\leq 2R_{n}$, by Theorem 1 of [33],

$$\begin{array}{c} P\left(|\sum_{b=1}^{B}W_{b,i,j}^{+}|>\tilde{n}x-C_{n}\right)≲\exp\left\{-\frac{C{\tilde{n}}^{2}x^{2}}{BR_{n}^{2}+R_{n}\tilde{n}x\left(\log B\right)\left(\log\log B\right)}\right\} \end{array}$$

for any $x>CB^{-1/2}S^{-1}$. Thus, we can conclude that

$$\begin{array}{c} \max_{i,j\in [p]}{\tilde{n}}^{-1}\left|I_{1,i,j}\right|=\max_{i,j\in [p]}|\frac{1}{\tilde{n}}\sum_{b=1}^{B}W_{b,i,j}|=O_{p}\left[\frac{{\left\{\log\left(pn\right)\right\}}^{3/2}}{B^{1/2}}\right] \end{array}$$

provided that $\log\left(pn\right)=o[\min\left\{S^{1/2},B{\left(\log n\log\log n\right)}^{-2}\right\}]$. By Bonferroni inequality and Theorem 1 of [32], we know that

$$\begin{array}{cc} P\left(\max_{i,j\in [p]}{\tilde{n}}^{-1}\left|I_{2,i,j}\right|>x\right)≲ & \;p^{2}\max_{i\in [p]}P\left(|\sum_{t=1}^{\tilde{n}}{\overset{˚}{Z}}_{t,i}|>CS^{-1/2}nx^{1/2}\right)\\ ≲ & \;p^{2}n\exp\left(-CS^{-1/3}n^{2/3}x^{1/3}\right)+p^{2}\exp\left(-CS^{-1}nx\right) \end{array}$$

for any $x\gg Sn^{-2}$. Then, we can conclude that $\max_{i,j\in [p]}{\tilde{n}}^{-1}\left|I_{2,i,j}\right|=O_{p}\left\{B^{-1}\log\left(pn\right)\right\}$ provided that $\log\left(pn\right)=o\left(n^{1/2}\right)$. Finally, Equation (1.12b) of [30] yields

$$\begin{array}{cc} \; & \;|\sum_{b=1}^{B}E\left\{\left(\sum_{t\in I_{b}}{\overset{˚}{Z}}_{t,i}\right)\left(\sum_{t\in I_{b}}{\overset{˚}{Z}}_{t,j}\right)\right\}-E\left\{\left(\sum_{t=1}^{\tilde{n}}{\overset{˚}{Z}}_{t,i}\right)\left(\sum_{t=1}^{\tilde{n}}{\overset{˚}{Z}}_{t,j}\right)\right\}|\\ \leq & \;\sum_{b=1}^{B-1}\sum_{\kappa =1}^{B-b}|\sum_{t_{1}\in I_{b}}\sum_{t_{2}\in I_{b+\kappa }}E\left({\overset{˚}{Z}}_{t_{1},i}{\overset{˚}{Z}}_{t_{2},j}\right)|≲\sum_{b=1}^{B-1}\sum_{\kappa =1}^{B-b}\sum_{\delta =1}^{B}\delta \exp\left[-C\left\{\delta +\left(\kappa -1\right)S\right\}\right]\\ ≲ & \;\sum_{b=1}^{B-1}\sum_{\delta =1}^{B}\delta \exp\left(-C\delta \right)+\sum_{b=1}^{B-1}\sum_{\kappa =2}^{B-b}B^{2}\exp\left\{-C\left(\kappa -1\right)S\right\}≲B+B^{3}\exp\left(-CS\right)≲B, \end{array}$$

which implies $\max_{i,j\in [p]}{\tilde{n}}^{-1}\left|I_{3,i,j}\right|=O\left(S^{-1}\right)$. Thus,

(A36) $$\begin{array}{c} \Gamma =O_{p}\left[\frac{{\left\{\log\left(pn\right)\right\}}^{3/2}}{B^{1/2}}+\frac{1}{S}\right] \end{array}$$

provided that $\log\left(pn\right)=o[\min\left\{S^{1/2},B{\left(\log n\log\log n\right)}^{-2}\right\}]$. It holds that

$$\begin{array}{c} \underset{x\in R}{\sup}\left|P\left({\hat{T}}_{n}^{G}\leq x\;|\;E\right)-P\left(T_{n}^{G}\leq x\right)\right|≲\Gamma^{1/2}\log p=o_{p}\left(1\right) \end{array}$$

provided that $\log\left(pn\right)=o\left[n^{\min\{(1-\vartheta )/2,\vartheta /7\}}\right]$. The proof of the second result of Theorem 2 under Case4 is the same as in the proof of the second result of Theorem 2 under Case3. Thus, we complete the proof of Theorem 2. □

## Appendix D. Proof of Theorem 3

**Proof.** Let $s=C\pi_{n}p^{2}S^{-1/2}$ in Case3 and $s=C\pi_{n}\left[B^{-1/2}{\left\{\log\left(pn\right)\right\}}^{3/2}+CS^{-1}\right]$ in Case4, where $\pi_{n}>0$ diverges at a sufficiently slow rate. Then s=o(1) provided that $p=o(S^{1/4})$ in Case3 and $\log\left(pn\right)=o\left(B^{1/3}\right)$ in Case4. Define an event

$$\Phi \left(s\right)=\left\{\max_{j\in [p]}|\frac{{\left({\hat{\Xi }}_{\tilde{n}}\right)}_{j,j}}{{\left(\Xi_{\tilde{n}}\right)}_{j,j}}-1|\leq s\right\},$$

where ${\hat{\Xi }}_{\tilde{n}}$ and $\Xi_{\tilde{n}}$ are specified in (A27) and (3), respectively. By (A34) and (A36) in Appendix C, we have

$$\begin{array}{cc} \max_{j\in [p]}\left|{\left({\hat{\Xi }}_{\tilde{n}}\right)}_{j,j}-{\left(\Xi_{\tilde{n}}\right)}_{j,j}\right|\leq & \;|{\hat{\Xi }}_{\tilde{n}}-\Xi_{\tilde{n}}{|}_{\infty }=o_{p}\left(s\right) \end{array}$$

holds under Case3 and Case4 with $\log\left(pn\right)=o\left(S^{1/2}\right)$. By Assumption 1(iii) and Assumption 2(iii), we know that $\min_{j\in [p]}{\left(\Xi_{\tilde{n}}\right)}_{j,j}>c$ holds under Case3 and Case4. Therefore,

$$\max_{j\in [p]}|\frac{{\left({\hat{\Xi }}_{\tilde{n}}\right)}_{j,j}}{{\left(\Xi_{\tilde{n}}\right)}_{j,j}}-1|\leq \frac{\max_{j\in [p]}\left|{\left({\hat{\Xi }}_{\tilde{n}}\right)}_{j,j}-{\left(\Xi_{\tilde{n}}\right)}_{j,j}\right|}{\min_{j\in [p]}{\left(\Xi_{\tilde{n}}\right)}_{j,j}}=o_{p}\left(s\right).$$

Then it holds that $P\left\{\Phi^{c}\left(s\right)\;|\;E\right\}=o_{p}\left(1\right)$ under Case3 and Case4. Let $\varrho =\max_{j\in [p]}{\left(\Xi_{\tilde{n}}\right)}_{j,j}$. Restricted on Φ(s), there exists a constant $C_{0}>0$ such that

$$\begin{array}{cc} E({\hat{T}}_{n}^{G}\;|\;E)\leq & \;C_{0}{\left(\log p\right)}^{1/2}\max_{j\in [p]}{\left({\hat{\Xi }}_{\tilde{n}}\right)}_{j,j}^{1/2}\leq {\left(1+s\right)}^{1/2}C_{0}{\left(\log p\right)}^{1/2}\varrho^{1/2}. \end{array}$$

By Borell inequality for Gaussian process,

$$\begin{array}{c} P\left\{{\hat{T}}_{n}^{G}>E\left({\hat{T}}_{n}^{G}\;|\;E\right)+x\;|\;E\right\}\leq 2\exp\left\{-\frac{x^{2}}{2\max_{j\in [p]}{\left({\hat{\Xi }}_{\tilde{n}}\right)}_{j,j}}\right\} \end{array}$$

for any x>0. Let $x_{0}=\varrho^{1/2}{\left(1+s\right)}^{1/2}\left[C_{0}{\left(\log p\right)}^{1/2}+{\left\{2\log\left(4/\alpha \right)\right\}}^{1/2}\right]$. Restricted on Φ(s), we have

$$x_{0}\geq E\left({\hat{T}}_{n}^{G}\;|\;E\right)+{\left(2\varrho \right)}^{1/2}{\left(1+s\right)}^{1/2}\log^{1/2}\left(\frac{4}{\alpha }\right),$$

which implies

$$\begin{array}{c} P\left\{{\hat{T}}_{n}^{G}>x_{0}\;,\Phi \left(s\right)\;|\;E\right\}\leq 2\exp\left\{-\frac{2\varrho (1+s)\log(4/\alpha )}{2\varrho (1+s)}\right\}=\frac{\alpha }{2}. \end{array}$$

Since $P\left\{\Phi^{c}\left(s\right)\;|\;E\right\}=o_{p}\left(1\right)$, then $P\{\Phi^{c}\left(s\right)\;|\;E\}\leq \alpha /4$ with probability approaching one. Hence, $P({\hat{T}}_{n}^{G}>x_{0}\;|\;E)\leq \alpha$ with probability approaching one. Similar to the proof of (A1), we know that ϱ≤C under Case3 and Case4. By the definition of ${\hat{\mathrm{cv}}}_{\alpha }$, it holds with probability approaching one that

$$\begin{array}{cc} {\hat{\mathrm{cv}}}_{\alpha }\leq & \;\varrho^{1/2}{\left(1+s\right)}^{1/2}\left[C_{0}{\left(\log p\right)}^{1/2}+{\left\{2\log\left(4/\alpha \right)\right\}}^{1/2}\right]≲{\left(\log p\right)}^{1/2} \end{array}$$

under Case3 with $p=o(S^{1/4})$ and Case4 with $\log\left(pn\right)=o\left(B^{1/3}\wedge S^{1/2}\right)$. Let $\mu_{X}={\left(\mu_{X,1},\ldots ,\mu_{X,p}\right)}^{T}=E\left(X_{t}\right)$, $\mu_{Y}={\left(\mu_{Y,1},\ldots ,\mu_{Y,p}\right)}^{T}=E\left(Y_{t}\right)$ and $j_{0}=\arg\max_{j\in [p]}\left|\mu_{X,j}-\mu_{Y,j}\right|$, then

$$\begin{array}{cc} T_{n}= & \;\sqrt{\frac{n_{1}n_{2}}{n_{1}+n_{2}}}{\left|{\hat{\mu }}_{X}-{\hat{\mu }}_{Y}\right|}_{\infty }\geq \sqrt{\frac{n_{1}n_{2}}{n_{1}+n_{2}}}|\frac{1}{n_{1}}\sum_{t=1}^{n_{1}}X_{t,j_{0}}-\frac{1}{n_{2}}\sum_{t=1}^{n_{2}}Y_{t,j_{0}}|\\ = & \;\sqrt{\frac{n_{1}n_{2}}{n_{1}+n_{2}}}|\frac{1}{n_{1}}\sum_{t=1}^{n_{1}}\left(X_{t,j_{0}}-\mu_{X,j_{0}}\right)-\frac{1}{n_{2}}\sum_{t=1}^{n_{2}}\left(Y_{t,j_{0}}-\mu_{Y,j_{0}}\right)+\mu_{X,j_{0}}-\mu_{Y,j_{0}}|\\ \geq & \;\sqrt{\frac{n_{1}n_{2}}{n_{1}+n_{2}}}\left|\mu_{X,j_{0}}-\mu_{Y,j_{0}}\right|-\sqrt{\frac{n_{1}n_{2}}{n_{1}+n_{2}}}|\frac{1}{n_{1}}\sum_{t=1}^{n_{1}}\left(X_{t,j_{0}}-\mu_{X,j_{0}}\right)-\frac{1}{n_{2}}\sum_{t=1}^{n_{2}}\left(Y_{t,j_{0}}-\mu_{Y,j_{0}}\right)|. \end{array}$$

Similar to the proof of (A1), we know that

$$|\frac{1}{n_{1}}\sum_{t=1}^{n_{1}}\left(X_{t,j_{0}}-\mu_{X,j_{0}}\right)-\frac{1}{n_{2}}\sum_{t=1}^{n_{2}}\left(Y_{t,j_{0}}-\mu_{Y,j_{0}}\right)|=O_{p}\left(n^{-1/2}\right)$$

under Case3 and Case4. If $|\mu_{X,j_{0}}-\mu_{Y,j_{0}}|\gg n^{-1/2}$, we can conclude that $P(T_{n}\geq C^{*}n^{1/2}|\mu_{X,j_{0}}-\mu_{Y,j_{0}}|)\to 1$ as n→∞ for some constant $C^{*}>0$. Due to ${\hat{\mathrm{cv}}}_{\alpha }≲{\left(\log p\right)}^{1/2}=o(n^{1/2}|\mu_{X,j_{0}}-\mu_{Y,j_{0}}\left|\right)$ under Case3 and Case4, we have that Theorem 3 holds under Case3 and Case4. □

## Appendix E. Additional Simulation Results

**Table A1:** The Type I error rates, expressed as percentages, were calculated by independently generated sequences ${\left\{X_{t}\right\}}_{t=1}^{n_{1}}$ and ${\left\{Y_{t}\right\}}_{t=1}^{n_{2}}$ based on (M2). The simulations were replicated 1000 times.

($n_{1},n_{2}$)	ρ	p	Yang	Dempster	BS	SD	CLX
(200,220)	0	50	4.6	2.3	6.4	4.5	3
		200	3.3	0	6.7	5.8	3.4
		400	3.7	0	5	4.4	4.1
		800	3.3	0	6.4	5.2	4.2
	0.1	50	6.2	12.4	23.4	18.4	9.8
		200	5.2	1.8	43.2	39.5	12.9
		400	5.8	0.1	63	59.7	14.7
		800	5.3	0	88.7	87.3	19.4
	0.2	50	8.1	35.6	51.3	44.3	21.9
		200	7.7	23.5	87.2	85.5	37.9
		400	9.2	16.9	98.5	98.3	43
		800	9.6	9.8	100	100	54.5
(400,420)	0	50	4.9	1.8	5.4	3.6	3.5
		200	3.5	0	6.4	5.3	3.2
		400	5	0	5.7	4.8	4.4
		800	4.3	0	6	5	4.5
	0.1	50	6.9	12.2	21.7	17	9.3
		200	4.9	1.9	41.7	39	11.1
		400	7.8	0.1	63.6	61.4	17.7
		800	7.3	0	87.9	86.7	18.3
	0.2	50	8.6	33.7	46.9	40.7	20.6
		200	7.7	23.7	86.3	84.7	31
		400	9.4	17.1	99.2	99	43.6
		800	9	9.5	100	100	53.2

**Table A2:** The Type I error rates, expressed as percentages, were calculated by independently generated sequences ${\left\{X_{t}\right\}}_{t=1}^{n_{1}}$ and ${\left\{Y_{t}\right\}}_{t=1}^{n_{2}}$ based on (M3). The simulations were replicated 1000 times.

($n_{1},n_{2}$)	ρ	p	Yang	Dempster	BS	SD	CLX
(200,220)	0	50	5.7	16.8	7.7	3	1.6
		200	4.3	14.9	6.9	0.9	1.6
		400	3.5	14.7	7.7	0.2	1.2
		800	4.2	15.4	6.9	0.2	1.7
	0.1	50	7.9	25.2	13.7	5.5	5.4
		200	6.2	23	12	2.7	5
		400	5.5	23.3	12.5	1.2	4.2
		800	6.9	24	12.9	0.7	5.5
	0.2	50	8.6	33.8	21	10.7	12.8
		200	7.5	32.5	19.7	5.8	13.9
		400	6.9	30.4	20.2	4.4	15
		800	9.3	32.3	20.7	1.7	18.4
(400,420)	0	50	5.4	13.9	6.7	1.7	1.6
		200	5.1	15.5	6.4	1	1.1
		400	5.3	14.1	7.1	0.8	1.3
		800	4	16.2	6.3	0.1	1.3
	0.1	50	6.9	21.3	10.7	4.7	5.4
		200	6.6	23.1	12.5	2.7	4.9
		400	7.3	22	11.4	1.7	5.9
		800	6.2	23.8	12.7	0.6	5.4
	0.2	50	8.2	31.8	18.2	8.6	11
		200	8.2	31	19.6	5.2	13.5
		400	8.7	32.6	18.9	3.9	14.7
		800	7.4	35.2	21.3	1.8	17.3
